# Genetic and phenotypic associations between peduncle characteristics and spike productivity in wheat under drought and normal conditions

**DOI:** 10.1007/s00122-025-05140-2

**Published:** 2026-01-13

**Authors:** Ahmed Sallam, Mostafa Hashem, Asmaa A. M. Ahmed, Saleh M. Ismail, P. Stephen Baenziger, Andreas Börner

**Affiliations:** 1 School of Biotechnology, Badr University in Assit (BUA), Assiut, Egypt; 2 Department of Genetics, Faculty of Agriculture, Assiut University, Assiut, 71526 Egypt; 3 Department of Genebank, Leibniz Institute of Plant Genetics and Crop Plant Research (IPK), Gatersleben, Germany; 4 Soils and Water Department, Faculty of Agriculture, Assiut University, Assiut, Egypt; 5 Department of Agronomy & Horticulture, University of Nebraska-Lincoln, Lincoln, NE 68583 USA

## Abstract

The peduncle stem plays an essential role in resource allocation and water transport to spike grains under normal conditions. Therefore, exploring peduncle traits and their relationships with spike production under drought stress may provide insights into the mechanisms that mitigate the effects of drought stress on grain yield in wheat. To address this challenge, a panel of 198 highly homozygous and diverse spring wheat varieties was evaluated under normal and drought conditions across two growing seasons. Peduncle traits, namely, length (PL), diameter (PD), and weight (PW), as well as spike traits, namely, spike length (SPL), number of spikelets/spike (NSPS), grain number/spike (GNPS), and grain yield per spike (GYPS), and thousand-kernel weight (TKW), were assessed. We revealed that PW and PD, unlike PL, were significantly and strongly associated with spike traits and grain weight under both conditions. Genome-wide association study (GWAS) revealed that spike and peduncle traits were controlled by different genetic mechanisms, as no stable markers were shared between these two groups. Distinct SNPs found between genotypes with contrasting peduncle traits led to the identification of a key SNP marker in a gene model encoding a protein highly expressed in the peduncle and spike of wheat. Comparing cultivars with low peduncle trait values to cultivars with high peduncle trait values, particularly PW and PD, high peduncle trait cultivars had greater yield-related trait values under both drought and normal conditions. The results of this study shed light on the importance of peduncle traits in enhancing wheat spike productivity under normal and drought stress conditions.

**Supplementary Information:**

The online version contains supplementary material available at 10.1007/s00122-025-05140-2.

## Introduction

Wheat (*Triticum* *aestivum*) production and productivity in dry environments have been the focus of extensive research efforts; however, drought tolerance remains a complex trait, with many adaptive mechanisms still not fully understood. At the reproduction growth stage, drought tolerance was investigated by estimating the stress susceptibility index, reduction due to drought stress (%), or selecting high-performance genotypes under dry environments (Sallam et al. 2019). Drought escape, drought avoidance, and drought tolerance are the better-defined mechanisms in plants. Exploring new adaptive traits/mechanisms is very important for expanding our knowledge of how plants alleviate the effects of drought stress, so that new cultivars with high drought tolerance can be bred. Improving new adaptive traits is very important for breeding programs that aim to select superior genotypes for production and crossing (Mourad et al. 2021).

Plant height in wheat is one of the most important yield-related traits that are included in many breeding programs because it correlates with other important traits, such as lodging and grain yield (Würschum et al. 2015). Plant height is routinely and frequently scored in any field experiment. Very few studies have reported the relationship between stem characteristics and grain yield in wheat (Ahmed et al. 2014; Niu et al. 2022; Feng et al. 2024; Wang et al. 2025). Sallam et al. (2015) reported a significant correlation between stem characteristics (second internode diameter, main stem weight, and stem density) and grain yield per spike in 21 F1 and 28 F2 populations produced from a diallel cross without selves and without reciprocal crosses of seven wheat genotypes under drought (D) and combined drought and heat (D + H) stresses. Additionally, the second internode diameter (from the soil) was significantly correlated with the thousand-kernel weight under D and D + H.

Carbohydrate translocation to the grain is an important process that determines final grain weight, but this process is affected by drought stress (Kobata et al. 1992). Carbohydrates can be obtained from post-anthesis photosynthesis, but the carbohydrates are stored temporarily in the stem before being remobilized to the grains (Kobata et al. 1992). Additionally, carbohydrates can also be obtained from pre-anthesis photosynthesis, where the carbohydrates are stored primarily in the stem and then remobilized to the grain during the grain-filling stage. When wheat plants are exposed to drought stress, a sharp decline in photosynthesis occurs, which leads to a reduction in grain assimilates, causing a significant decrease in grain weight (Kobata et al. 1992). Flag leaf photosynthesis alone cannot support grains with carbohydrates, and a considerable amount of carbohydrates for grain is needed and must come from reserves assimilated and stored in the stem (Wardlaw 1990).

The peduncle is the stem from the internode below the spike of wheat to the spike, and it has been reported that the peduncle is an important trait for controlling photosynthetic efficiency (Wang et al. 2023.), fungal disease resistance, and lodging resistance (Madic et al. 2016). However, this part of the stem may play other important roles, especially under abiotic stresses (e.g., drought and heat stresses), as it is the part of the stem that is directly connected to the spike (Gebbing 2003a, b). The vascular system in the peduncle plays a critical role in transporting assimilates to grains (Wardlaw 1965). Under drought stress, peduncle stems retain high amounts of potential water and nutrients compared with flag leaves. Wardlaw (1965) reported that stems have many advantages over flag leaves because of their anatomical, ultrastructural, and physiological structures for controlling photosynthesis. Gebbing (2003a, b) was previously reported that the high stomatal density found in the peduncle provided important adaptive mechanisms to the ecological environment during all the grain-filling stages. Hence, investigating the genetic variation in peduncle characteristics and their relationships with spike traits under abiotic stress may shed light on important mechanisms that alleviate the effects of drought stress in wheat.

Genetic variation in peduncle length has been investigated only in dry environments and rain-fed environments by and in well-watered environments (Liu et al. 2023). The correlations between PL and yield traits have been poorly studied. Looking at other important characteristics, such as stem diameter and weight, which have not been previously reported, may also be useful to expand the understanding of adaptive mechanisms under drought stress in wheat.

Genome-wide association studies (GWAS) are still considered one of the most important types of association analysis for identifying alleles and genes associated with target traits. GWAS have been widely used to identify markers associated with spike traits in wheat. However, only one study reported markers associated with peduncle length in wheat under drought stress (Rahimi et al. 2019). Very few studies have performed GWAS for peduncle length under drought and normal conditions in wheat (Wang et al. 2023). Therefore, a considerable gap in understanding the genetic control of peduncle traits in wheat remains.

The objectives of this study were to investigate the genetic variation in peduncle traits, explore their associations with spike traits under normal and drought stress conditions, and elucidate the genetic control of peduncle traits by identifying promising SNP markers for further utilization in MAS to improve drought tolerance in wheat.

## Materials and methods

### Plant material

A set of 198 highly diverse spring wheat genotypes representing a wide range of diverse agronomic and physiological traits were selected for the study (Supplementary Table S1). These genotypes were collected from 22 different countries obtained from the United States Department of Agriculture. The number of genotypes used from each country is shown in Supplementary Table S1.

### Experimental layout

**Fig. 1:**
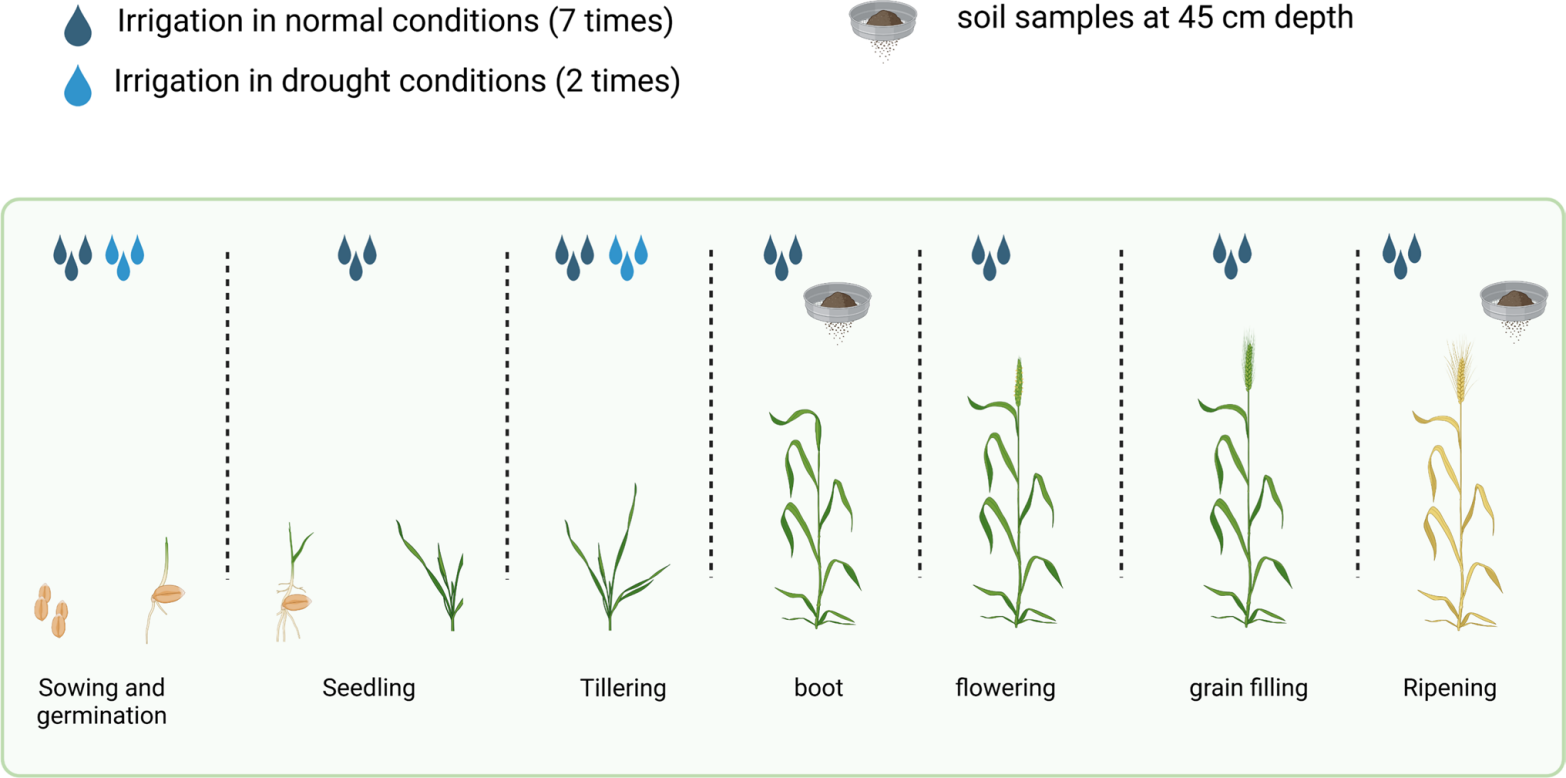
The number of irrigations in normal and drought conditions, and soil sampling time through the two seasons

All the genotypes were sown in two consecutive seasons, 2020 and 2021, under normal (N) and drought (D) conditions at the Experimental Field Station of the Department of Genetics, Assiut, Egypt (27°11′20.36′′N, 31°10′06.45′′E), where the soil is clay loam. Each condition was considered as an environment: N2020, D2020, N2021, and D2021. A randomized complete block design (RCBD) with two replications was used. In both seasons and conditions, the seeds of each genotype were sown in one 1.5 m row, with 10 cm between seeds within a row and 10 cm between rows. The genotypes under N and D conditions were planted on the normal wheat sowing date. Under normal conditions, all the genotypes received planting irrigation and seven irrigations throughout the growing season, whereas under drought stress, the plants received two irrigations; at the sowing date and when the majority of lines were at the tillering stage (Fig. 1).

### Soil moisture content

Soil moisture content was measured using the gravimetric method as described in Holliday (1990). The soil moisture content was measured in the upper soil layer with a 45 cm depth. The measurements were done two times during the growing season, before the earliest line headed and after the latest line headed. Six soil samples were randomly selected from each experimental soil area of the two studied scenarios (fully irrigated and stress condition). Once the soil samples were collected, they were put in weighted moisture canes, and the wet weight of the soil samples of each scenario was recorded. Then, the soil samples were transferred to the laboratory and dried in the oven at 110 degrees Celsius until constant weight, and the dry weight of each soil sample was recorded. The gravimetric soil moisture content was calculated by dividing the loss in weight (sample wet weight—sample dry weight) by the sample dry weight. To convert the gravimetric moisture content to volumetric water content (Vol.%), the gravimetric moisture content was multiplied by soil bulk density, which was 1.2 g/cm^3^ under the conditions of this experiment.

### Measured traits

In both seasons, ten traits were scored for each genotype under both conditions: The chlorophyll content (CC) was measured at the grain-filling stage via the chlorophyll content meter (Model CCM-200) of the flag leaves of each genotype (Opti-Sciences, Hudson, USA). The heading date (HD; days) was scored as the number of days from sowing to the date when 50% of the plants started heading. Five plants per replication were chosen to measure the following traits. Plant height (PH; cm) was scored from the ground to the tip of the main culms´ spike at maturity. The following spike traits were recorded for each genotype: main spike length (SL; cm), number of grains per spike (GNPS), number of spikelets per spike (NSPS), and grain yield per spike (GYPS, gm). After harvest, the peduncle traits of the main culm were recorded as follows: peduncle length (PL; cm) measured from the last internode of the main stem to the base of the spike, peduncle diameter (PD; mm) measured at the center of peduncle via digital calipers (0–150 mm), and peduncle weight (PW; gm) measured after harvest via a digital scale.

### Changes in a trait due to drought stress

For the spike and peduncle traits, the reduction due to drought stress in each genotype in the two seasons was calculated according to the following equation:

$${\text{RDD }} = \, \left( {{\mathrm{Xn}} - {\mathrm{Xd}}} \right)/{\text{Xn }} \times {1}00$$

where Xn is the main average over the trait under normal conditions, and Xd is the main average of the same trait under drought stress.

### Statistical analyses of the phenotypic data

The statistical analysis of the phenotypic data was performed with PLABSTAT software (Utz 1997) using the following model:

$$Y_{{{\mathrm{iknj}}}} = \, \mu \, + y_{k} + t_{{\mathrm{n}}} + g_{{\mathrm{i}}} + r_{{\mathrm{j}}} + {\text{yg }} + {\text{ tg }} + {\text{ ytrg}}\left( {{\mathrm{error}}} \right)$$

where Y_ij_ is the observation of genotype *i* in year *k*, treatment *n,* and replication *j*; *μ* is the general mean; and *y*_*k*_, *t*_n_, *g*_i_ and *r*_j_ are the main effects of year, treatment, genotype, and replication, respectively. Years, genotypes, and replications were considered random effects, whereas treatment was considered a fixed effect. Broad-sense heritability (H^2^) estimates for each trait was estimated as the ratio of genotypic (*ơ*^2^_*g*_) to genotypic (*ơ*^2^_*p*_) variance for each trait via PLABSTAT via the HERT command.

### Selection index for drought tolerance

Three selection indices (Wedel 1962) were calculated to identify the best- and lowest-performing genotypes under drought stress. The spike index (SI) was used to better describe GYPS (*X*_1_) via two secondary traits, GNPS (*X*_2_) and NSPS (*X*_3_) as:

$${\text{SI }} = \, b_{1} X_{1} + \, b_{2} X_{2} + \, b_{3} X_{3}$$

The peduncle index (PI) was used to improve PW (*X*_1_) via the secondary trait PD (*X*_2_) as:

$${\text{PI }} = \, b_{1} X_{1} + \, b_{2} X_{2}$$

where *b*_1_, *b*_2_, and *b*_3_ (for GYPS) are the index coefficients. The vector of the Smith–Horsel index coefficient b is calculated as shown in Baker (2021). The drought index (DI) is calculated from the SI and PI as follows:

$${\text{DI }} = \, \raise.5ex\hbox{$\scriptstyle 1$}\kern-.1em/ \kern-.15em\lower.25ex\hbox{$\scriptstyle 2$} \, \left[ {\left( {{\mathrm{SI}}/{\mathrm{SD}}_{{{\mathrm{SI}}}} } \right) \, + \, \left( {{\mathrm{PI}}/{\mathrm{SD}}_{{{\mathrm{PI}}}} } \right)} \right]$$

where SD_SI_ and SD_PI_ are the phenotypic standard deviations of the SI and PI, respectively. High DI values identified the best genotypes were under drought stress.

The 20 genotypes with the highest DI values were selected each year, and the Venny tool (https://bioinfogp.cnb.csic.es/tools/venny/) was used to identify the common genotypes in both growing seasons under drought stress.

### Total-soluble carbohydrates (TSC)

The TSCs were analyzed in the peduncle of the selected genotypes (highest and lowest DI). The dried stem peduncle (0.05 g) was boiled in glass tubes containing 5 mL distilled water at 100 °C for two hours. The extract was then cooled and filtered, and the supernatant was kept in deep freeze until use. This extract was used for the estimation of TSC (mg/g DW) according to Fales (1951).

## Genetic analyses

### Genotyping and genome-wide association study

The wheat genotypes were previously genotyped via two different types of genotyping methods, as described by Sallam et al. (2024a, b) and as follows:

1. 25 K Infinium iSelect array (25 K set): All 197 tested genotypes were genotyped via the GmbH TraitGenetics Section, Gatersleben, Germany. The results of genotyping via the 25 K method revealed 21,093 SNPs after filtration.
2. Genotyping-by-sequencing (GBS set): Only 103 genotypes were genotyped via GBS methods by Mourad et al. (2020). A set of 11,362 SNP markers remained after marker and genotype filtration.

In both methods, the markers were filtered based on the criteria described by Alqudah et al. (2020) as follows: Heterozygous loci were excluded, followed by the exclusion001 of markers with more than 20% missing data and a minor allele frequency < 5%. Finally, genotypes with more than 20% missing data were excluded. As a result, 197 (25 K set) and 103 (GBS set) genotypes with 21,093 (25 K) and 11,362 (GBS) markers, respectively, were used for GWAS.

The 25 K and GBS sets presented a clear population structure according to Sallam et al. (2024a) and Mourad et al. (2020), respectively. In this study, genome-wide association analysis was performed for all traits scored in the two growing seasons and under both conditions as described in detail by Sallam et al. (2024a). The GWAS was performed via nine different models via the memory-efficient, visualization-enhanced, and parallel-accelerated (rMVP) package: general linear model (GLM) + PCA, GLM + Kinship, GLM + PCA + Kinship, mixed linear model (MLM) + PCA, MLM + Kinship, MLM + PCA + Kinship, fixed and random model circulating probability unification (FarmCPU) + PCA, FarmCPU + Kinship, and FarmCPU + PCA + Kinship (Yin et al. 2021). The best GWAS model for each trait was determined according to the distribution of the expected and observed p-values in the quantile‒quantile plot (Q‒Q plot) (Alqudaha et al. 2019). In Q–Q plot, the nonsignificant markers were positioned on the diagonal line, except the significant markers, which were above the diagonal line. Two significant thresholds; a *p*-value ≤ 0.001 (− log10 > 3.00) and suggestive *p*-values (1/*N* (number of markers) (Li et al. 2012) were used to identify significant markers.

### Gene annotation

The gene annotations for stable markers, which were significantly associated with the same traits under each condition in the two growing seasons, were investigated via the Ensembl Plants database for *Triticum aestivum* (https://plants.ensembl.org/Triticum_aestivum/Info/Index). The International Wheat Genome Sequencing Consortium (IWGSC) Reference Sequence v1.0 was used to determine the physical positions of the SNPs resulting from GBS. On the other hand, the flanking sequences of the SNP markers from the 25 K set were obtained from the GrainGenes database (https://wheat.pw.usda.gov/GG3/). The physical positions (GBS set) and flanking sequences (25 K set) were then blasted against the Ensembl database (https://plants.ensembl.org/Triticum_aestivum/Info/Index) to identify candidate genes and their functional annotations. Candidate genes were selected if the significant SNPs were located within them.

## Results

### Soil moisture content

A single-factor analysis of the soil moisture content under both conditions is presented in Supplementary Table S2. Highly significant differences in soil moisture were observed in the two growing seasons before and after the heading growth stages. Before harvest, the differences in soil moisture were significant in 2020/2021 and highly significant in 2021/2022. Drought stress was more severe in the first season than in the second season.

### Effects of drought stress on peduncle and spike traits

On average, drought stress has a negative impact on all traits, in both seasons (Supplementary Fig. 1). All the traits were reduced due to drought stress except for TKW in 2020, which was slightly greater under drought conditions than under normal conditions (0.02%). Notably, for all traits except TKW, the reduction due to drought (RDD) was greater in the first season than in the second. The reduction in chlorophyll content due to drought stress was 7.15 and 3.61 in 2020 and 2021, respectively. In both seasons, all spike traits were greatly reduced due to drought stress. Among the spike traits, the greatest RDD was found for GNPS in 2020 and SPL in 2021. NSPS was the spike trait least affected by drought stress, NSPS presented the lowest reduction, with 2.56% and 1.64% in 2020 and 2021, respectively. Among the peduncle traits, PW presented the greatest reduction in both years, with 23.7% and 20.4% in 2020 and 2021, respectively, followed by PD and PL. The plant height decreased by 16.7% and 9.5% in 2020 and 2021, respectively. A percentage (5.79% of all genotypes) flowered earlier under drought than did the control in 2020, whereas in 2021, 2.36% of all genotypes flowered earlier under drought than did the control.

### Genetic variation in the peduncle and yield traits under drought stress

The results of the combined ANOVA of the studied traits are presented in Table 1. There were highly significant differences between the years in all traits except SPL and GYPS. Significant height differences were found among the genotypes between the treatments, *G* × *Y*, and *G* × *T* for all the traits. High *H*^2^ estimates were found for all the traits, ranging from 0.92 (CC) to 0.99 (HD).

**Table 1:** Analysis of variance F-values and broad-sense heritability (H^2^) for all traits under both conditions, and drought indices under drought conditions in two seasons (2020/2021 & 2021/2022)

	CC	HD	PH	SPL	PL	PD	PW	NSPS	GNPS	GYPS	TKW
Years (Y)	1280.36**	741.83**	27.72**	2.17	219.98**	100.23**	97.65**	3.91*	5.71*	0.24	3.66 +
Treatment (T)	25.35**	497.75**	885.14**	105.42**	386.05**	491.21**	385.35**	105.26**	59.97**	54.33**	2.06
Replicates (R)	1.16	56.18**	1.32	5.66*	0.57	74.48**	12.16**	2.04	4.21*	3.45 +	0.04
Genotypes (G)	13.19**	117.86**	35.26**	23.98**	18.48**	18.47**	17.66**	17.13**	18.46**	23.58**	37.78**
G × Y	2.78**	4.11**	2.70**	2.57**	2.71**	1.68**	2.43**	1.93**	2.78**	2.44**	2.25**
G × T	2.10**	1.99**	1.61**	2.82**	1.45**	1.54**	1.68**	1.57**	1.97**	2.09**	1.44**
H^2^	92.42	99.15	97.16	95.83	94.59	94.59	94.34	94.16	94.58	95.76	97.35

Drought indices						
	2020/2021			2021/2022		
	SI	PI	DI	SI	PI	DI
R	1.53	4.63**	5.62**	41.47**	51.52**	68.10**
G	8.78**	5.81**	8.89**	12.91**	6.35**	10.71**
H^2^	88.61	82.79	88.75	92.25	8.25	90.66

^*^, ** refer to the significant level at 0.05 and 0.01 significantly

CC, chlorophyll content; HD, heading date; PH, plant height; SPL, spike length; PL, peduncle length; PW, peduncle weight; PD, peduncle diameter; NSPS, number of spikelet per spike; GNPS, grain number of spike; GYPS, grain yield per spike; TKW, thousand-kernel weight; SI, drought spike index; PI, drought peduncle index; DI, drought index including SI and PI

The analysis of variance for each year is presented in Supplementary Tables 3 and 4. A highly significant difference was found between the two treatments in each season for all measured traits. Moreover, highly significant variation among all the genotypes for all the studied traits was observed. The ANOVA revealed high genetic variation among the genotypes for all three selection indices in each year under drought stress (Table 1). Highly significant variation in the drought indices was observed among all the genotypes. All indices presented high heritability (*H*^2^) in both years (Supplementary Tables 5 and 6).

The minimum, maximum, and mean values for all trait measurements in the two seasons for all the genotypes under normal and drought conditions are presented in Table 2. The three drought indices had lower values in 2021 than in 2020.

**Table 2:** Minimum (Min), maximum (Max), and mean of each trait scored in the study under normal (N) and drought stress conditions (D) in 2020/2021 and 2021/2022

	2020/2021						2021/2022					
	Normal			Drought			Normal			Drought		
	min	max	mean	min	max	mean	min	max	mean	min	max	mean
CC	10.1	36.4	19.06	16.17	39.89	26.86	9.68	51.58	27.87	10.1	29.54	17.69
HD	88	150	110.27	79.5	124	97.84	77	129	100.21	89	133.5	103.63
PH	68	179	127.77	63.33	147.66	114.76	61.33	170.67	126.81	54.75	143.75	106.33
SPL	3	32	12.97	4.33	21.16	11.52	4.67	26.67	12.72	6	20.25	11.65
PL	18.5	68	46.14	14.5	50.83	35.55	15.67	62.33	39.54	19.5	59.75	39.71
PD	2.13	5.24	3.47	2	4.39	2.91	1.95	5.65	3.24	2.08	4.31	3.03
PW	0.18	1.72	0.66	0.11	1	0.45	0.12	1.74	0.56	0.12	1.22	0.51
NSPS	19	32	26.18	20	30.67	25.43	19	33.67	25.85	19	30.5	24.73
GNPS	12	146	63.1	31.67	121.17	60.08	29.67	149	63.28	18	106.5	55.92
GYPS	0.43	7.9	2.79	0.82	6.07	2.55	0.77	7.77	2.78	0.32	5.3	2.48
TKW	22.78	65.70	43.45	24.50	82.50	44.44	17.80	66.33	44.26	22.73	69.60	42.73
SI	–	–	–	0.74	5.73	2.877	–	–	–	0.77	5.92	2.479
PI	–	–	–	0.09	1.01	0.414	–	–	–	0.1	0.85	0.391
DI	–	–	–	1.5	7.07	3.24	–	–	–	0.9	5.97	2.869

CC, chlorophyll content; HD, heading date; PH, plant height; SPL, spike length; PL, peduncle length; PW, peduncle weight; PD, peduncle diameter; NSPS, number of spikelet per spike; GNPS, grain number of spike; GYPS, grain yield per spike; TKW, thousand-kernel weight; SI, drought spike index; PI, drought peduncle index; DI, drought index including SI and PI

**Fig. 2:**
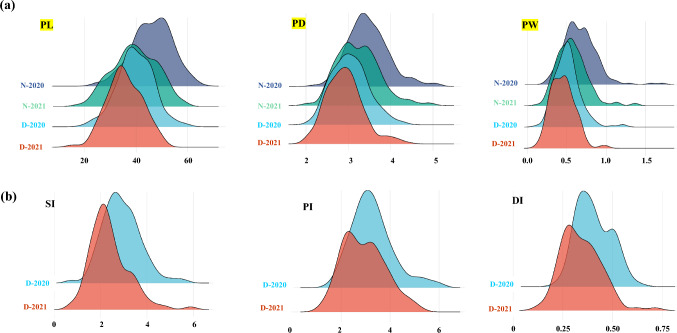
**a** Density diagram for all genotypes in peduncle traits under normal and drought conditions in the two growing seasons (2020 and 2021), **b** three drought indices

The distributions of peduncle traits, as well as drought indices, for all the genotypes in the four environments are presented in Fig. 2, while the density plot for the rest of the traits across the four environments is presented in Supplementary Fig. 2.

### Phenotypic and genotypic correlations among measured traits

The phenotypic correlations among all traits under normal and drought conditions in 2020 and 2021 are presented in Tables 3 and 4, respectively.

**Table 3:** Phenotypic correlation for normal conditions (normal font, above the diagonal) and drought conditions (bold font, below the diagonal) among all traits scored in 2020

	CC	HD	PH	SPL	PL	PD	PW	NSPS	GNSP	GYPS	TKW
CC	–	− 0.429**	− 0.410**	0.011	− 0.366**	0.235**	− 0.078	− 0.047	0.097	0.228**	0.298**
HD	− 0.400**	–	0.406**	0.196**	− 0.09	− 0.322**	− 0.215**	0.336**	− 0.487**	− 0.639**	− 0.601**
PH	− 0.354**	0.185**	–	0.033	0.535**	− 0.172*	0.328**	0.12	− 0.349**	− 0.350**	− 0.206**
SPL	**0.026**	0.261**	**0.008**	–	− 0.055	0.212**	0.096	0.219**	− 0.048	− 0.123	− 0.085
PL	− 0.271**	**0.004**	0.773**	**− 0.044**	–	0.135	0.662**	− 0.095	0.018	0.048	0.078
PD	0.374**	− 0.380**	**− 0.038**	0.140*	**0.115**	–	0.625**	0.184*	0.417**	0.490**	0.377**
PW	0.209**	− 0.339**	0.376**	**0.094**	0.528**	0.715**	–	0.053	0.235**	0.347**	0.362**
NSPS	**− 0.051**	0.225**	**0.005**	0.218**	**− 0.026**	0.176*	**0.063**	–	0.160*	− 0.013	− 0.342**
GNSP	**− 0.071**	**− 0.071**	**− 0.045**	**− 0.063**	**0.04**	0.211**	**0.138**	0.350**	–	0.860**	0.264**
GYPS	0.150*	− 0.290**	**− 0.047**	**0.01**	**− 0.008**	0.419**	0.355**	0.198**	0.772**	–	0.655**
TKW	0.361**	− 0.444**	**0.012**	**0.052**	**− 0.004**	0.446**	0.439**	− 0.218**	**− 0.077**	0.481**	ـــ
SI	0.166*	− 0.356**	**− 0.107**	**0.027**	**− 0.004**	0.441**	0.357**	0.278**	0.793**	0.999**	0.448**
PI	0.153*	− 0.358**	0.357**	0.185**	0.591**	0.711**	1.000**	**0.129**	0.157*	0.349**	0.412**
DI	0.194**	− 0.434**	0.151*	**0.129**	0.356**	0.699**	0.823**	0.247**	0.578**	0.819**	0.522**

Bold font represents the drought treatment

^*^, ** refer to the significant level at 0.05 and 0.01 significantly

CC, chlorophyll content; HD, heading date; PH, plant height; SPL, spike length; PL, peduncle length; PW, peduncle weight; PD, peduncle diameter; NSPS, number of spikelet per spike; GNPS, grain number of spike; GYPS, grain yield per spike; TKW, thousand-kernel weight; SI, drought spike index; PI, drought peduncle index; DI, drought index including SI and PI

**Table 4:** Phenotypic correlation for normal conditions (normal font, above the diagonal) and drought conditions (bold font, below the diagonal) among all traits scored in 2021

	CC	HD	PH	SPL	PL	PD	PW	NSPS	GNPS	GYPS	TKW
CC	–	− 0.450**	− 0.311**	− 0.014	0.073	0.297**	0.242**	− 0.142*	0.307**	0.393**	0.330**
HD	0.147*	–	0.217**	0.246**	− 0.462**	− 0.473**	− 0.508**	0.327**	− 0.579**	− 0.689**	− 0.504**
PH	− 0.224**	**0.135**	–	0.043	0.574**	0.076	0.351**	0.289**	− 0.187**	− 0.165*	− 0.083
SPL	0.148*	**0.126**	**0.059**	–	− 0.259**	0.083	− 0.026	0.293**	− 0.065	− 0.092	− 0.045
PL	− 0.291**	− 0.357**	0.680**	**− 0.095**	–	0.405**	0.704**	− 0.001	0.194**	0.228**	0.152*
PD	**0.067**	− 0.647**	**− 0.028**	**0.131**	0.332**	–	0.779**	0.247**	0.495**	0.565**	0.406**
PW	**− 0.095**	− 0.605**	0.290**	**0.072**	0.665**	0.778**	–	0.112	0.396**	0.513**	0.441**
NSPS	**0.025**	0.152*	0.190**	0.178*	**0.088**	0.249**	0.162*	–	0.180*	− 0.057	− 0.335**
GNPS	**0.047**	− 0.691**	− 0.153*	**0.003**	0.220**	0.717**	0.574**	0.262**	–	0.817**	0.240**
GYPS	**0.099**	− 0.795**	− 0.160*	**0.018**	0.215**	0.768**	0.681**	**0.111**	0.860**	–	0.716**
TKW	**0.122**	− 0.513**	**− 0.108**	**0.046**	**0.097**	0.475**	0.502**	− 0.155*	0.246**	0.665**	–
SI	**0.137**	− 0.716**	− 0.249**	0.160*	0.172*	0.783**	0.686**	0.160*	0.866**	1.000**	0.670**
PI	**− 0.068**	− 0.566**	0.261**	0.228**	0.646**	0.807**	1.000**	0.230**	0.583**	0.693**	0.511**
DI	**0.037**	− 0.697**	**0.006**	0.211**	0.444**	0.864**	0.916**	0.212**	0.788**	0.920**	0.642**

Bold font represents the drought treatment

^*^, ** refer to the significant level at 0.05 and 0.01 significantly

CC, chlorophyll content; HD, heading date; PH, plant height; SPL, spike length; PL, peduncle length; PW, peduncle weight; PD, peduncle diameter; NSPS, number of spikelet per spike; GNPS, grain number of spike; GYPS, grain yield per spike; TKW, thousand-kernel weight; SI, drought spike index; PI, drought peduncle index; DI, drought index including SI and PI

In the four environments (N-2020, N-2021, D-2020, and D-2021), CC was significantly and negatively correlated with HD. Heading date was negatively correlated with PH, PD, and PW in the four environments and positively correlated with GYPS. PD was positively and significantly associated with NSPS, GNPS, GYPS, and TKW in the four environments. Similarly, PW was significantly and positively correlated with GNPS, GYPS, and TKW in the four environments. PL did not show stable, significant correlations with spike traits across environments. Among the peduncle traits, PW was highly significantly correlated with PL and PD across the four environments. PL was significantly correlated with PD only in 2021 under both conditions. Weak and nonstable correlations were found between SPL and peduncle traits. The genetic correlations among all traits in each environment are presented in Supplementary Tables 7 and 8. Notably, the genetic correlations among all the traits were greater than the phenotypic correlations under both conditions in the two seasons.

**Fig. 3:**
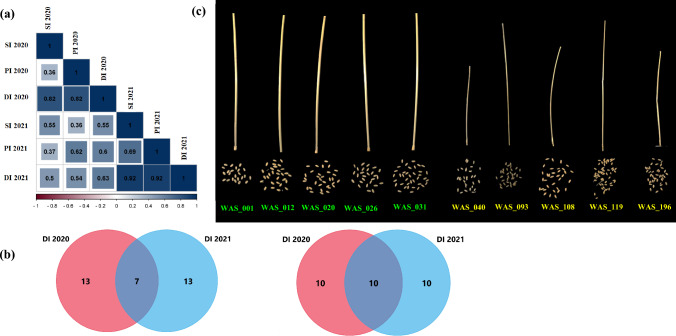
**a** Phenotypic correlation between the drought indices in 2020 and 2021, **b** the best and lowest 20 genotypes under drought stress and common genotypes between the two seasons, **c** differences between top high (left) and low (right) genotypes for peduncle traits

Under drought stress in both growing seasons, highly significant correlations were found between the spike index (SI) and all peduncle traits in both growing seasons under drought stress. The peduncle index (PI) was also positively and significantly correlated with spike traits. The drought indices, including SI and PI, had highly significant and positive correlations with all spike traits except SPL and all peduncle traits. Moreover, highly significant correlations were found among the three indices in each year and between the two years (Fig. 3a).

The correlations between the reduction due to drought stress (RDD) in the peduncle and spike traits are presented in Table 5. In both growing seasons, RDD in PD and PW was positively and highly correlated with RDD in NSPS, GNPS, GYPS, and TKW. The RDD in PL had positive and significant correlations with only GNPS and GYPS.

**Table 5:** Correlation between the reduction in peduncle and spike traits due to drought stress in 2020/2021 and 2021/2022

Trait	**2020**		**2021**				
	PL	PD	PW		PL	PD	PW
SPL	0.084	0.1756*	− 0.028		0.058	0.357**	0.285**
NSPS	0.123	0.507**	0.383**		0.197*	0.458**	0.433**
GNPS	0.302**	0.460**	0.510**		0.284**	0.467**	0.325**
GYPS	0.248**	0.273**	0.342**		0.251**	0.483**	0.402**
TKW	0.098	0.186**	0.177*		− 0.170*	0.073	0.189**

^*^, ** refer to the significant level at 0.05 and 0.01 significantly

SPL, spike length; NSPS, number of spikelet per spike; GNPS, grain number of spike; GYPS, grain yield per spike; TKW, thousand-kernel weight; PL, peduncle length; PW, peduncle weight; PD, peduncle diameter

### Identifying the best- and lowest-performing genotypes under drought

As DI, including PI and SI, was highly significantly correlated with spike and peduncle traits under drought stress in the two growing seasons, all the genotypes were sorted on the basis of their SI values (high values indicated high performance under drought stress). Then, the 20 highest and 20 lowest values were selected each year. In both years, a total of seven wheat genotypes were among the 20 genotypes with the highest DI values in both years (Fig. 3b), whereas ten genotypes presented the lowest DI values (Fig. 3b, Supplementary Table S9). Among the seven genotypes, Giza-36 was determined to be one of the highest-performing genotypes under drought stress and presented the highest yield and peduncle traits. OK91G158 was determined to be the genotype with the lowest peduncle trait and productivity. The differences in peduncle and grain traits between the five genotypes with the highest DI values and those with the lowest DI values are presented in Fig. 3c. Compared with those with low peduncle traits, those with high peduncle traits presented greater spike traits.

**Fig. 4:**
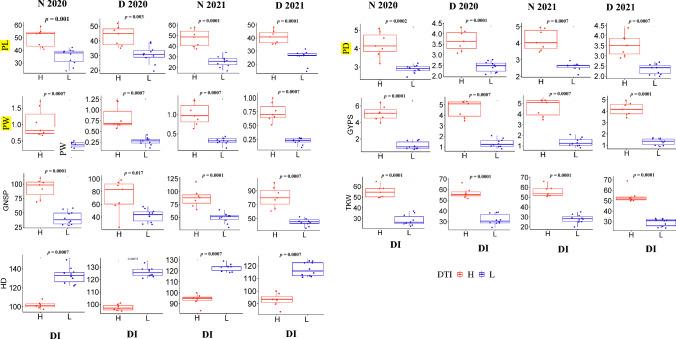
Box plot shows the performance of both the highest and lowest genotypes in each morphological traits under both conditions (normal, drought stress) and in the two growing seasons (2020 and 2021)

**Fig. 5:**
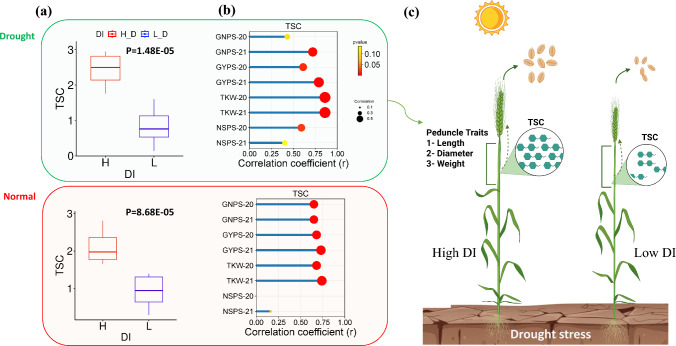
**a** Box plot showing the differences between genotypes with the highest and lowest DI under normal and drought conditions, **b** correlation between TSC and spike traits under normal and drought conditions, **c** the role of peduncle traits in supporting spike traits under drought stress

On the basis of the two groups of genotypes (high DI vs low DI), the differences in all traits between these two groups were tested (Fig. 4). Interestingly, highly significant differences were found in all traits except SPL, CC, and PH under the four environments between the two contracting groups. Interestingly, the total-soluble carbohydrates (TSC) were analyzed in the stem peduncle in the two contracting groups based on DI. Highly significant variation was found between the two groups under drought and normal conditions (Fig. 5a). Highly significant correlations were found between TSC and GYPS, TKW, and GNPS (except in D_2020) under the four environments (N_2020, D_2020, N_2021, and D_2021). The nonsignificant correlation was observed between TSC and NSPS (except D_2020).

### Genome-wide association study (GWAS)

**Fig. 6:**
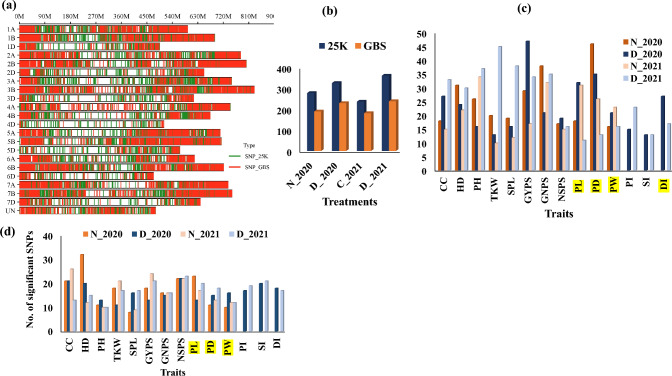
**a** Distribution of SNP markers resulted from 25 K (21,093) and GBS (11,363) across the different wheat chromosomes, **b** the total number of significant SNPs (25 K and GBS) detected under control and drought conditions in the two seasons (2020 and 2021), **c, d** the total number of significant SNPs (25 K, GBS) associated with each trait

A GWAS was performed for all traits scored in this study under the four environments. The distributions of SNP markers generated from 25 K and GBS on each chromosome and genome are presented in Fig. 6a and Supplementary Fig. 3, respectively. The number of significant SNPs detected under drought stress was greater than that detected under normal conditions from the 25 K genotyping method, whereas the opposite was true for the GBS genotyping methods. The number of significant markers detected for each trait in each environment is presented in Fig. 6b. The detailed GWAS results for all traits scored under drought and normal conditions are presented in Supplementary Tables 10 and 11, respectively. On the basis of the results of the QQ plot, the correct GWAS model was selected. FarmCPU + PCA + kin was the best-fitting model for most of the traits scored under the two conditions in each season (Supplementary Fig. 4a–n).

**Fig. 7:**
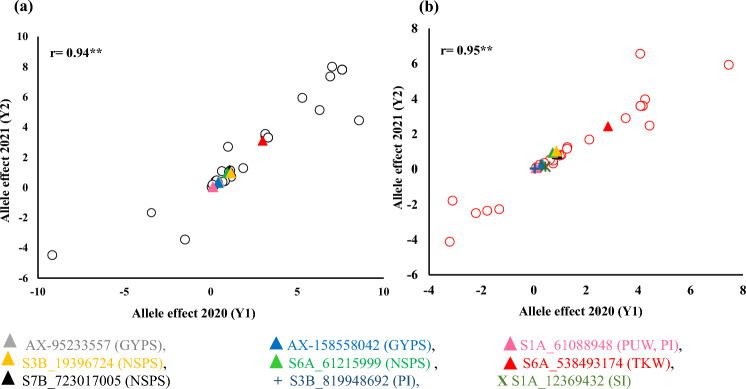
The allele effect of all common markers in both growing seasons (2020–2021) under both conditions **a** control and **b** drought

GWAS identified 26 (25 K) and 39 (GBS) significant markers, which were either shared among different traits or consistently associated with the same trait under identical environmental conditions (drought or normal) and/or across multiple environments (Supplementary Table S12). Most of these markers were located on chromosome 3B. A total of 32 and 34 stable markers were found to be associated with the same trait under normal and drought conditions, respectively. Interestingly, six markers were found to be associated with the same trait in the four environments: GYPS (two), PW (one), NSPS (two), and TKW (one) (Supplementary Table S13). The target alleles of these six markers had the same effect on the trait in all environments under both conditions. For example, allele A of the SNP marker AX-95233557 was associated with increased GYPS in all environments in both growing seasons, whereas the C allele of the SNP marker S1A_61088948 was associated with increased PW in all environments in both years. To confirm the stability of the allele effect of the significant markers associated with the same trait under each condition and across all environments, the correlation of allele effects in the two years was calculated (Fig. 7). The target allele effects of the stable markers associated with the same trait in 2020 were highly and significantly correlated with the effects of the same allele in 2021 under normal (*r* = 0.94**) and drought stress (0.95**) conditions.

Notably, the common significant markers associated with the same traits were also associated with other yield traits. For example, the SNP marker AX-111638065 was found to be associated with HD under drought stress in both growing seasons: HD under N-2021, PW (D-2021), PD (D-2020), and GYPS (D-2021).

None of the significantly stable markers were shared between spike traits and peduncle traits. Stable markers for the selection of the three selection indices (PI, SI, and DSI), which were calculated under only drought stress in each year, were investigated (Supplementary Table S14). One SNP marker (S1A_12369432) was associated with the SI in both years under drought stress, whereas two stable SNP markers (S1A_61088948 and S3B_819948692) were associated with the PI under drought stress in both years. However, no shared stable markers were found between the PI and the SI.

The gene annotations of the stable significant markers were investigated (Supplementary Table S14). Among the 65 SNPs, 27 were located within 30 gene models, encoding 22 functional proteins and five hypothetical proteins. Among these 22 functional proteins, 11 strongly correlated with drought tolerance in wheat.

Notably, some of the significant markers identified in this study were previously reported in earlier studies under drought stress conditions in wheat (Supplementary Table S15). The SNP marker CAP8_rep_c4857_90, which was associated with the NSPS in this study, was found to be associated with awn length, the harvest index, and leaf area under drought stress according to Qaseem et al. (2018).

The target allele of each significant marker was determined in each selected genotype having high DI values (Supplementary Table S16). Omara-007 had the highest number of target alleles (368) detected all four environments, while PI525241 had the lowest number of target alleles (281).

### SNP signals differentiating the tolerant and susceptible genotypes

**Fig. 8:**
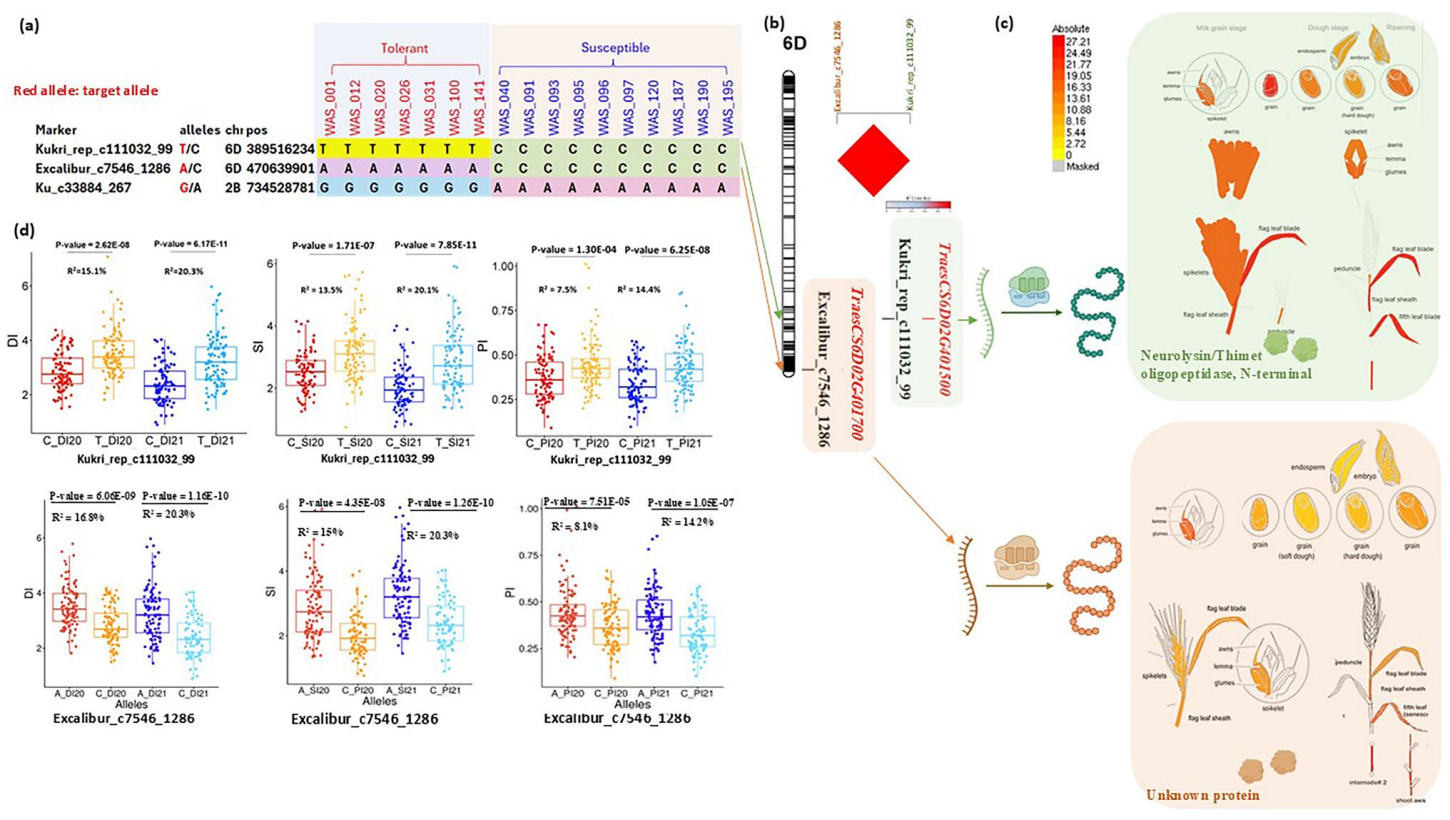
**a** Distinct SNPs between genotypes with the highest and lowest DI (including PI and SI); **b** SNP positions and LD on chromosome 6D; **c** expression of two genes on chromosome 6D in spike and stem peduncle; **d** allele group differences for two SNPs on chromosome 6D

The seven drought-tolerant and ten drought-susceptible genotypes, on the basis of DSI, were used to determine whether there were distinct SNPs between these two groups. The 25 K set was used for this purpose, as not all genotypes were genotyped via the GBS method. Among the 21,093 SNP markers (25 K), only three distinct SNP markers clearly (0.01%) differentiated the tolerant and susceptible genotypes (Fig. 8a). Two of these markers were located on the 6D chromosome, whereas the other marker was located on the 2A chromosome. Single-marker analysis was performed between the three markers and the SI, PI, and DI using all the genotypes (198). The analysis revealed that the three markers were highly and significantly associated with all three indices in both years (Supplementary Table S14). The markers were located within three different gene models that encode three different proteins. An investigation of the relationship between the functional protein of each gene and the corresponding trait revealed that the SNP marker Kukri_rep_c111032_99 and Excalibur_c7546_1286 (6D) were the most important markers that were significantly associated with the corresponding trait. Kukri_rep_c111032_99 marker was found to be located within TraesCS6D02G401500, which encodes a neurolysin/thimet oligopeptidase with an N-terminus. The gene was highly expressed in the peduncle and spike traits of wheat during the development stages (Fig. 8c). The biological process of this gene involves auxin transportation and auxin signals in wheat stems. Although the other two SNPs were also highly associated with the three indices, their gene and functional proteins did not provide evidence of an association with the spike or peduncle. Notably, the SNP markers Excalibur_c7546_1286 and Kukri_rep_c111032_99 were also detected by GWAS, with significant associations with DI in 2020 (Supplementary Table S10).

## Discussion

### Genetic variation in spikes and peduncles

Drought stress significantly reduced all the studied traits in both seasons. The reduction due to drought stress in all the studied traits in the first season was greater than the reduction in all the traits in the second season. Peduncle weight was the trait most affected by drought stress, with reduced values of 23.7% and 20.4% in the D-2020 and D-2021 seasons, respectively. On the other hand, drought stress had little effect on NSPS compared with all the other traits scored in the two years under drought stress. All the traits followed a normal distribution across the four environments. The analysis of variance revealed high genetic variation among all the genotypes for all the traits. This highly significant genetic variation made the detection of novel allele variants possible via GWAS. Moreover, the population included highly diverse wheat genotypes originating from 22 different countries, and high genetic variation in all the traits was expected. The same population was successfully used earlier to identify genes and markers associated with fungal disease resistance (Esmail et al. 2023), heavy metal tolerance (Mourad et al. 2024, 2025), salinity stress tolerance (Hasseb et al. 2022), and drought stress at the seedling stage (Sallam et al. 2024a, b). Significant differences in traits were found between the two treatments, indicating that drought stress was applied successfully. This can also be observed from differences in the soil moisture analyzed before anthesis and at or near maturity in both growing seasons. It was a successful treatment and that it accurately represented terminal drought. The broad-sense heritability was high for all the traits, indicating that selection for promising high-drought-tolerant wheat genotypes under drought stress is feasible. The significant differences in the genotype × treatment interaction indicated that the genotypes responded differently under drought and normal conditions, as would be expected (Chen et al. 2012; Bapela et al. 2022).

The phenotypic correlation between spike and peduncle traits provides very valuable and novel information on the critical role of the peduncle in supporting grain weight under drought stress. Among the peduncle traits scored in this study, PW and PD had highly significant and positive correlations with GNPS, GYPS, and TKW under normal and drought conditions in both growing seasons (four environments). In contrast, PL exhibited weak or nonsignificant correlations with GNPS, GYPS, and TKW in the four environments. This clearly highlights the importance of peduncle weight and diameter, rather than length, in increasing grain weight and number under both conditions. The higher density of stomata in the peduncle may play a significant role in improving photosynthetic efficiency by increasing the surface area for gas exchange, regulating water loss through transpiration, and supporting grain filling in the late stages (Kong et al. 2010). Therefore, on the basis of our results of this study, a wider peduncle may indicate greater vascular capacity or greater stem reserves, which could support enhanced photosynthesis and nutrient accumulation and assimilate transportation or remobilization to the grain during the grain-filling stage. The advantages of having a wider peduncle can be extended by improving water transportation and minimizing water loss through stomatal density under drought stress. Kong et al. (2010) analyzed the anatomical traits of wheat peduncles during grain development in Jimai 22 (a winter wheat genotype). They reported that the peduncle has important anatomical, ultrastructural, and physiological characteristics compared with the flag leaf and that these important advantages play a critical role in grain filling. These characteristics regulate phosphoenolpyruvate carboxylase, which is important for controlling carbon assimilation activity and supplying substrates for carbohydrate synthesis during grain filling in the late growth stage. This can be noted the analysis of TSC in the selected genotypes based on drought index (highest and lowest genotypes in DI). Genotypes with distinct peduncle characteristics showed higher levels of TSC compared to those with lower TSC. The strong, significant, and positive correlation between TSC and spike traits—especially GYPS, GNPS, and TKW (Fig. 5b)—further confirms the role of peduncle characteristics in supporting spike development under both normal and drought conditions. Therefore, a thicker peduncle can assimilate more TSC, which is subsequently transferred to the spike during the grain-filling stage (Fig. 5c).

Notably, in our study, only PW and PD were found to have stable and significant positive correlations with HD across the four environments, indicating that earlier flowering genotypes generally were higher in PD and PW. In addition, Kong et al. 2010 described the role of stomatal density in peduncles in increasing photosynthesis efficiency and water use efficiency. Under drought stress, photosynthesis can be impaired due to stomatal closure; therefore, genotypes with high PD and PW may overcome this problem because of their high stomatal density compared with those with low PD and PW. This conclusion can be observed from this study, as the correlation between HD and both PW and PD under drought stress was greater than that under normal conditions in both years. These results further support the importance of PW and PD in improving yield traits over PL (Tables 3 and 4). Peduncle weight (PW) and diameter (PD) are more important than peduncle length (PL) because they reflect vascular capacity. Thicker and heavier peduncles provide greater xylem and phloem area for water and assimilate transport, as well as storage reserves that can be remobilized under stress (Huo and Kassab 2009). In contrast, longer peduncles do not necessarily enhance transport and may increase resistance, explaining why PUW and PUD were more strongly linked to yield under drought.

PL has been reported to play a role in and be associated with plant height, wheat pathogen resistance, and lodging resistance (Liu et al. 2023). In this study, highly positive and significant stable correlations were found between PL and PH in all four environments. Wang et al. (2023) studied the variation in PL under normal and drought conditions in a set of 282 wheat genotypes. However, the associations between peduncle length and yield traits were not reported or investigated in these studies. In a rain-fed environment, Rahimi et al. (2019) did not find any significant correlation between PL and yield traits (grain number per spike, grain yield, TKW) in a set of 298 Iranian genotypes. Although genetic variation in PL has also been studied by Liu et al. (2023) and Wang et al. (2023), its relationship with yield traits has not been investigated, and Liu et al. (2023) said that the relationship between PL and final-grain wheat is still unclear. All these studies are in agreement with the results reported here with respect to the correlation between PL and yield traits. In this study, PL was found to be negatively and significantly correlated with CC under drought stress (in both years) and normal conditions (in the 2020–2021 growing season). A negative and significant correlation was found between these two traits under normal conditions by Yadav et al. (2023). However, different correlations between these two traits have been reported in earlier studies. The correlation between CC and PL was found to be nonsignificant under normal conditions (Khalid et al. 2023), significant under normal conditions (Javed et al. 2022), and nonsignificant under drought conditions (Javed et al. 2022).

The highly significant correlation between the reduction in peduncle traits and spike traits due to drought stress highlights the importance of the relationship between peduncle and spike traits, especially under drought stress, in both years. The greater the reduction in peduncle traits, especially PD and PW, was, the greater the reduction in NSPS, GNPS, and GYPS. The smaller the reduction in peduncle traits, especially PD and PW, was, the smaller the reduction in NSPS, GNPS, and GYPS. Notably, there was no stable significant correlation between either PW or PD and the NSPS. Moreover, no promising significant correlation was found between a reduction in peduncle traits (PL, PW, and PD) due to drought stress and a reduction in TKW. This difference could be attributed mainly to the differences in the genotypes’ responses. Notably, the genotypes with high PW and PD presented either a small reduction in TKW or a slight increase in TKW. Notably, the reduction in PL due to drought stress was significantly and positively correlated with GNPS and GYPS. This result may shed light on the importance of how much the length of the peduncle is reduced rather than the absolute peduncle length under specific conditions. A greater reduction in PL may lead to a significant decrease in starch, vascular bundles, and carbohydrates stored in the stem, hence reducing grain weight. Moreover, this may also explain the nonstable correlation found between PL and other yield traits. Therefore, calculating the reduction in each genotype due to drought stress for the respective traits also provides valuable information on the relationships among these traits. Hence, the ideal genotype may exhibit a small reduction in PW, PD, and PL under drought stress, as it is expected that the same genotype will show a small reduction in yield traits, thereby increasing the production of the final yield. These stable and consistent phenotypic correlations are important for providing new insights into the role of peduncle characteristics, for the first time, in enhancing key yield traits under normal and drought conditions in both growing seasons.

### Selection of promising wheat genotypes under drought stress

To select the most promising high-yielding genotypes under drought stress, three selection indices were created: SI (including spike traits), PI (including peduncle traits), and DI (including SI and PI). The selection index, which includes many traits, is better than single-trait selection and integrates genetic and economic considerations into a single framework (Baker 2021). Despite the complexity of the calculations and efforts required to estimate the three indices, such indices provide a potent tool for discriminating genotypes with high spike and peduncle traits under drought stress (Sallam et al. 2018).

The correlations between PI and spike traits and between SI and peduncle traits, as well as the strong significant correlations between DI and both spike and peduncle traits, confirmed the strong relationships between peduncle and spike traits under drought stress. The highly significant correlation between DI in 2020 and 2021 (*r* = 0.92**) made the selection feasible. On the basis of the DI, the most promising high-performance (high spike and peduncle traits) genotypes were selected each year, resulting in seven genotypes that presented high spike and peduncle traits in both growing seasons under drought stress. Out of the seven genotypes, Sohag-5 was previously reported as a drought-tolerant genotype at the seedling stage (Ahmed et al. 2022). To further investigate this relationship (spike and peduncle), the lowest-performing genotypes were also selected (ten genotypes). The comparison of yield between the two groups of genotypes evaluated in this study under both conditions and across the 2 years revealed consistent and highly significant differences in all traits except PH, SPL, and CC. The high-performing genotypes (high DI values) presented very high PL, PW, PD, GNSP, GYPS, and TKW values compared with the yield traits of the ten lowest-performing genotypes (low DI values). Moreover, the seven highly selected genotypes flowered earlier than those in the other groups did. These results confirmed the correlation between peduncle and spike traits found across all 198 genotypes.

### Genetic analyses for spike and peduncle traits

#### Genome-wide association study

In this study, SNP markers produced via two different genotyping methods were used for GWAS. The SNP array (25 K) provides high accuracy, known marker positions, and reliable data for a fixed set of SNPs. More importantly, the majority of these SNPs fall within gene models, making the SNP array method ideal for gene identification, target analyses, or comparisons across studies (Geethanjali et al. 2024). The GBS method, on the other hand, offers high marker density and the ability to discover novel SNPs and/or genomic regions not covered by the SNP array, providing additional insights into genetic diversity and greater detection of important genes associated with target traits. Together, both methods can enhance the resolution and robustness of GWAS. According to marker and genotype filtration, two sets were produced, namely, the 25 K and GBS sets, which included 198 and 103 genotypes, respectively. It has been reported that 100–500 individuals are needed for performing GWAS (Alqudah et al. 2020). The sets (25 K and GBS) were individually used to identify alleles and genes associated with salinity stress tolerance, alkaline-saline tolerance, heavy metal tolerance, fungal disease resistance, and drought tolerance at the seedling stage via GWAS (Esmail et al. 2023).

The GWAS in this study revealed a total of 2243 significant SNPs associated with yield traits under normal and drought conditions in both growing seasons (four environments). The total number of SNPs varied by environment. The environment has a significant effect on SNP detection in GWAS, especially when analyzed traits are controlled by many genes related to drought tolerance, spikes, and peduncle traits (Eltaher et al. 2021). The marker‒trait associations were detected at a *P*-value > 0.001, which is widely used in GWAS. Using stringent p-values, such as those derived from the Bonferroni correction or false discovery rate, may lead to the loss of important markers/genes with minor effects. A *p*-value of 0.001 in GWAS serves as a relaxed yet reasonable threshold for identifying potential candidate associations. However, functional validation remains critical to confirm the true association. For example, Sallam et al. (2024a, b) validated one important SNP marker associated with leaf wilting at a threshold of *P* > 0.001 in the spring and winter populations at the seedling stage (Eltaher et al. 2021). Moreover, a SNP marker associated with recovery after drought stress was found in the winter association set at a *p*-value of < 0.001 and in the winter biparental population. As all the traits scored in this study are polygenic traits, identifying markers with major and minor effects is important for revealing the genetic control of peduncle and spike traits under both conditions. Interestingly, five significant markers that were detected at *p* < 0.001 were previously reported in different spring and winter wheat genetic backgrounds, with their associations with yield traits in wheat under drought stress (Supplementary Table S14).

In the current study, the GWAS analysis was performed using three different statistical models; GLM, MLM, and FarmCPU. PCA, Kinship, and PCA + Kinship were included in each model to correct the effect of population structure and avoid of spurious associations that resulted from population structure analysis. The spurious association is among the major problems affecting the efficiency of detecting marker–trait association in GWAS analysis (Larsson et al. 2013). In general, it is recommended to use all the available GWAS models to identify the best fit model for each trait and marker set. The best GWAS model for each trait was determined according to the distribution of the expected and observed p-values in the quantile‒quantile plot (Q‒Q plot). In Q–Q plot, the nonsignificant markers were positioned on the diagonal line except the significant markers which were above the diagonal line. The marker‒trait associations were detected at a *P*-value > 0.001, which is widely used in GWAS. Using stringent *p*-values, such as those derived from the Bonferroni correction or false discovery rate, may lead to the loss of important markers/genes with minor effects. A *p*-value of 0.001 in GWAS serves as a relaxed yet reasonable threshold for identifying potential candidate associations. However, functional validation remains critical to confirm the true association. For example, Sallam et al., (2024a, b) validated one important SNP marker associated with leaf wilting at a threshold of *P* > 0.001 in the spring and winter populations at the seedling stage (Eltaher et al. 2021). Moreover, a SNP marker associated with recovery after drought stress was found in the winter association set at a *p*-value of < 0.001 and in the winter biparental population. As all the traits scored in this study are polygenic traits, identifying markers with major and minor effects is important for revealing the genetic control of peduncle and spike traits under both conditions. Interestingly, five significant markers that were detected at *p* < 0.001 were previously reported in different spring and winter wheat genetic backgrounds, with their associations with yield traits in wheat under drought stress (Supplementary Table S14). The GWAS analysis revealed a total of 2243 significant SNPs associated with yield traits under normal and drought conditions in both growing seasons (four environments). The total number of SNPs varied by environment. The environment has a significant effect on SNP detection in GWAS, especially when analyzed traits are controlled by many genes related to drought tolerance, spikes, and peduncle traits (Eltaher et al. 2021).

In this study, stable significant markers that were found to be associated with the same trait under the same conditions in the two growing seasons as well as with the same trait under the four environments were prioritized for further analysis. The significant marker can be considered a validated marker if its effects remain significant across years, locations, or different populations (Sallam et al. 2023). Most of these stable markers were also found to be associated with other traits, indicating that these markers also have pleiotropic effects, indicating that these markers influence multiple traits simultaneously and would be very useful for marker-assisted selection after validation in different genetic backgrounds (Hashem et al. 2023). Notably, the effects of the stable markers on the trait, under normal or drought conditions or both conditions in the two years, were also consistent. This was observed from the high correlation found in the allele effects between the two years under normal (Fig. 6a) and drought stress (Fig. 6b). For example, the target C allele of the SNP marker S1A_61088948 was found to increase the PW under both conditions in the two seasons. In both years, the C allele of the SNP marker S6A_538493174 had the same allele effect on TKW, as it increased the traits by ~ 3.0 g under normal conditions, whereas it increased TKW by an average of 2.6 g under drought conditions. Such markers with stable and consistent effects on traits could be valuable markers for marker-assisted selection to accelerate the genetic improvement of grain yield per se and resilience to drought in molecular breeding programs for wheat. The stable allele effects indicate that these alleles can contribute positively to yield components. More importantly, they present minimal interaction with environmental factors, confirming their reliable performance across years and environments.

PW and PD were significantly associated with GYPS, TKW, and GNPS and stable shared markers were found between them. After the SI and PI were created, no markers were common between the two indices. Only one common stable marker was associated with DI, including PI and SI, in both growing seasons under drought stress. The use of the selection index in GWAS may help detect markers and genes for a group of traits when it is difficult to discover any marker for individual traits. This result indicated that although PW and PD enhanced yield traits under both conditions, it seems that spike and peduncle traits are controlled by different genetic mechanisms.

Interestingly, the results of GWAS were utilized to identify the number of targ**et al**leles of each significant SNP in the seven selected genotypes. WAS_020 (Omara-007) possessed the highest number of target alleles, with 368 alleles. The same genotype was found to possess nine drought tolerant genes (BIN1, NIM1, RHD2, OAT, OBF5, PEPR1, EDSI, DREB1-D, and DREB1-D2) (Sallam et al. 2024b). This result indicated that this genotype could be an important source of drought tolerance in wheat for future breeding programs to produce cultivars having high tolerance to drought stress.

### Gene annotation for promising and important markers under normal and drought conditions

The gene annotation in this study was performed for the most important SNP markers, which were divided into two groups: 1. SNP markers that were previously reported in earlier studies under drought stress and 2. stable markers that were significantly associated with the same traits under the same conditions in the two growing seasons or the four environments.

The SNP marker BS00064935_51 was found to be located within the TraesCS4B02G194900 (FAD7) gene model, which encodes an N-terminal fatty acid desaturase. FAD 7, which is localized in chloroplasts, plays an important role in maintaining thylakoid membrane function, ensuring efficient photosynthesis under drought conditions. Mutation of FAD 7 reportedly results in a 15% reduction in chlorophyll content of the mutant Arabidopsis plants compared with wild-type plants (McCourt et al. 1987). This SNP marker (BS00064935_51) was found to be associated with CC under drought stress and with plant height under drought in earlier studies. The allele effect of this SNP marker in CC was 2.297 (Supplementary Table S15). There was a highly significant negative correlation between CC and PH in the four environments in this study. The SNP marker CAP8_rep_c4857_90 was associated with NSPS under D_2021 in this study and with three traits, namely, awn length, harvest index, and leaf area, in a spring wheat population (European genotypes) under drought stress in the study of (Qaseem et al. 2018). This marker was found to be located in TraesCS7D02G377300, which encodes the vesicle transport protein Got1/SFT2-like. The relationship between this protein and drought tolerance in plants was not identified in earlier studies.

### Distinct SNPs between the highest- and lowest-performing wheat genotypes

Although GWAS is considered the most powerful tool for identifying linked SNPs with target traits, it has several limitations that affect the identification of important associated SNPs. GWAS do not account well for gene‒environment interactions, which affect many polygenic traits, such as drought tolerance (Eltaher et al. 2021). The same SNPs may have different effects under different environments, making confirming their true role difficult (Eltaher et al. 2021). Therefore, identifying genotypes with extreme phenotypes for a trait of interest, along with available SNP data, may help reveal important SNPs that GWAS might miss. In this context, the extremely contrasting genotypes for DI (Fig. 3b, Supplementary Table S9) and their SNP data generated from 25 K were used. Only three SNPs were clearly present in the seven genotypes with the highest DI values, and these SNPs were absent in the ten genotypes with the lowest DI values. Among these three markers, two, Excalibur_c7546_1286 and Kukri_rep_c111032_99, were located on the 6D chromosome within two gene models, TraesCS6D02G401500 and TraesCS6D02G401700, respectively. The two SNP markers were showed high significant LD (Fig. 8b), indicating that these two markers tend to be co-inherited together. The TraesCS6D02G401500 (TaOOP) gene model, which encodes Neurolysin/Thimet oligopeptidase, N-terminal. The ortholog of this protein in rice is encoded by the OsOOP gene, which is involved in the biological processes of auxin transport and auxin signaling (KnetMiner). The other gene model encodes to unknown protein. The transcriptomic wheat datasets of the two gene models (Wheat eFP Browser) confirmed the high expression of this protein (Neurolysin/Thimet oligopeptidase, N-termina) in both peduncle and spike traits, confirming the role of this gene in enhancing peduncle and spike traits together (Fig. 8c). Transcriptomic and gene expression databases such as the Wheat eFP Browser, a tool that provides spatial and temporal gene expression profiles across different wheat tissues, developmental stages, and environmental conditions, were used to confirm the results of genetic association analyses (Borrill et al. 2015, 2016). Single-marker analysis between these two markers (located on 6D) and the three selection indices (SI, PI, and DI) confirmed the presence of highly significant differences between the two groups of genotypes carrying different alleles (Fig. 8d, Supplementary Table 14). This marker was detected via GWAS in the first growing season but not in the second season. This confirmed the notion that genotype‒environment interactions play a crucial role in identifying important and stable SNPs via GWAS. Additionally, phenotypic data quality and distribution can significantly affect the power of relatedness correction models in GWAS, potentially leading to the absence of SNP markers in some environments (Korte and Farlow 2013). The other distinct SNPs, Ku_c33884_267 did not show any evidence associated with peduncle or spike traits under any conditions. The marker sequence (101 nucleotides long) was partially aligned with the TraesCS2A02G506800 gene model (67 nucleotides long). However, it might provide important and novel information. Further expression analysis experiments should be conducted for these three genes to validate their associations with peduncle traits before their use for marker-assisted selection. These three SNPs should be validated in different genetic backgrounds and also the expression the three gene models should be investigated. Distinct SNPs between genotypes with two different and extreme phenotypes may provide new insights into important genes and help overcome some limitations of GWAS, such as gene‒environment interactions. Further genetic experiments should be conducted to confirm these findings.

In conclusion, the results of this study shed light on novel important adaptive traits to drought stress. To the best of our knowledge, this is the first study reveals the association between the diameter and weight of the peduncle and spike traits under normal and drought conditions. The results revealed that PD and PW play a key role in mitigating the effects of drought stress and support grain and spike traits under normal and drought conditions. PL was not associated with spike traits or grain weight. However, selecting for high peduncle traits could be highly beneficial for improving wheat production and productivity under these conditions. Seven genotypes with high peduncle and spike traits could be used in further breeding programs to produce drought-tolerant cultivars. GWAS revealed that peduncle and spike traits are controlled by different genetic mechanisms. Distinct SNPs between genotypes with extreme and contrasting phenotypes may help overcome these limitations, particularly genotype‒environment interactions, in GWASs for identifying important SNPs associated with target traits.

## Supplementary Information

Below is the link to the electronic supplementary material.

Supplementary file1 (PPTX 46620 kb)

Supplementary file2 (XLSX 1498 kb)

## Data Availability

All phenotypic analyses are presented in the Supplementary files. The SNP datasets generated during and/or analyzed during the current study are not publicly available owing to their involvement in ongoing projects but are available from Prof. Dr. Ahmed Sallam upon reasonable request.
