# Supplementary material for: Genetic and phenotypic associations between peduncle characteristics and spike productivity in wheat under drought and normal conditions
Source: Theor Appl Genet. 2026 Jan 13;139(1):34. doi: 10.1007/s00122-025-05140-2 (PMC12799726; doi:10.1007/s00122-025-05140-2)
Supplement: Supplementary file 1 — Supplementary file1 (PPTX 46620 kb) [file 122_2025_5140_MOESM1_ESM.pptx]

## Slide 1
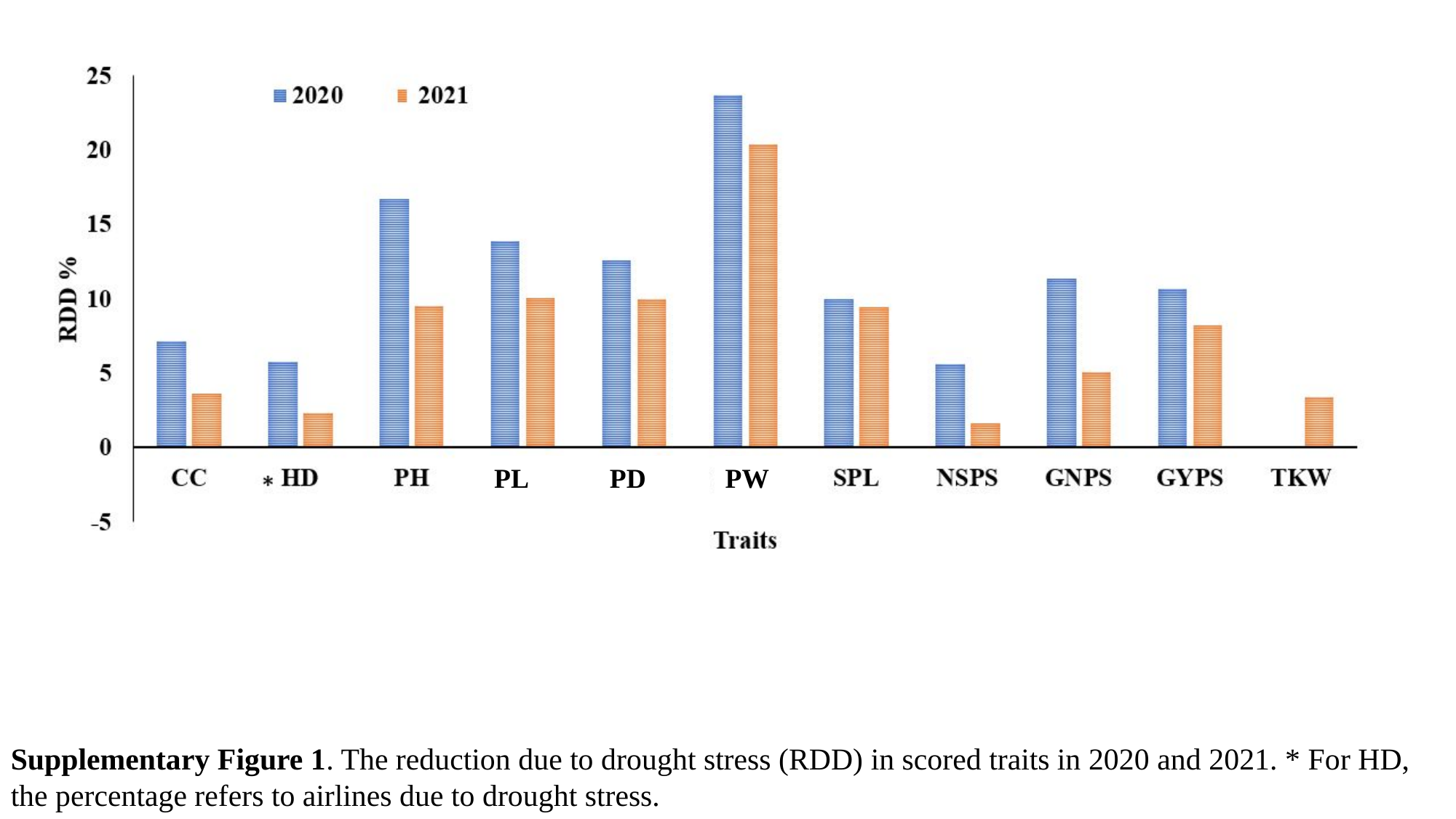

*
PL
PD
PW
Supplementary Figure 1. The reduction due to drought stress (RDD) in scored traits in 2020 and 2021. * For HD, the percentage refers to airlines due to drought stress.

## Slide 2
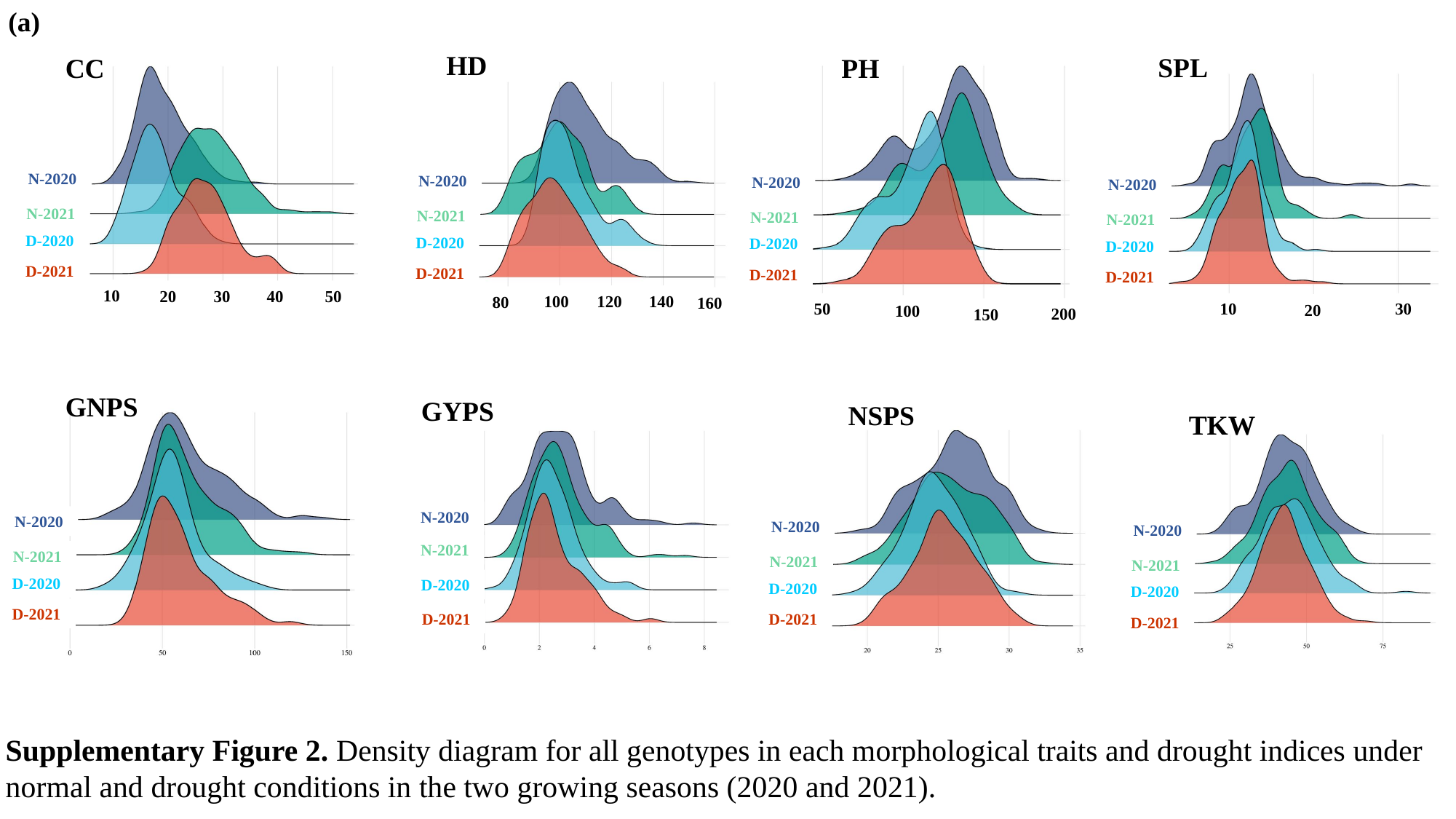

(a)
HD
SPL
PH
CC
N-2020
N-2020
N-2020
N-2020
N-2021
N-2021
N-2021
N-2021
D-2020
D-2020
D-2020
D-2020
D-2021
D-2021
D-2021
D-2021
10
20
30
40
50
100
120
140
80
160
50
10
30
20
100
200
150
GNPS
GYPS
NSPS
TKW
N-2020
N-2020
N-2020
N-2020
N-2021
N-2021
N-2021
N-2021
D-2020
D-2020
D-2020
D-2020
D-2021
D-2021
D-2021
D-2021
Supplementary Figure 2. Density diagram for all genotypes in each morphological traits and drought indices under normal and drought conditions in the two growing seasons (2020 and 2021).

## Slide 3
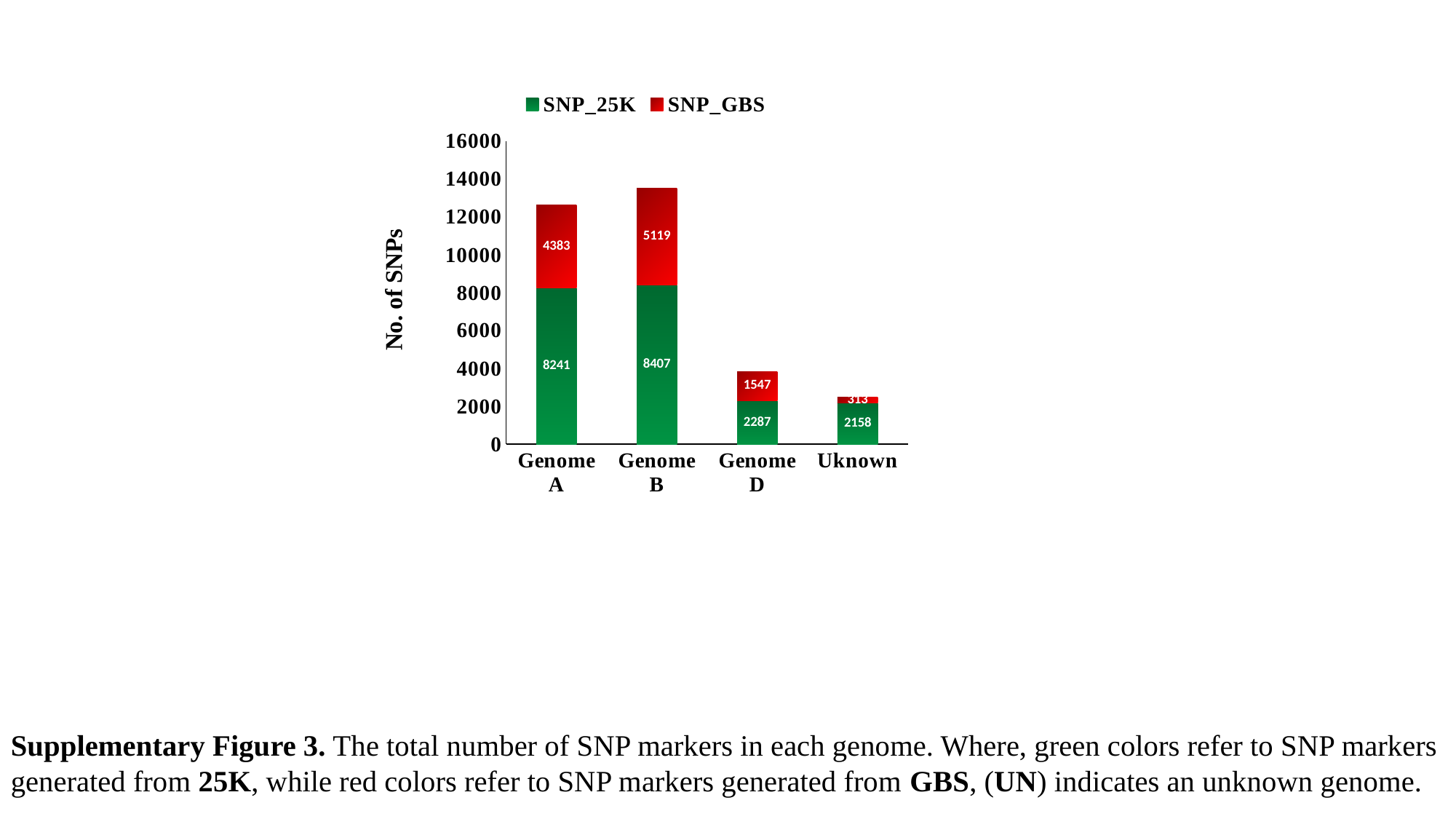

### Chart
| Category | SNP_25K | SNP_GBS |
|---|---|---|
| Genome A | 8241.0 | 4383.0 |
| Genome B | 8407.0 | 5119.0 |
| Genome D | 2287.0 | 1547.0 |
| Uknown | 2158.0 | 313.0 |Supplementary Figure 3. The total number of SNP markers in each genome. Where, green colors refer to SNP markers generated from 25K, while red colors refer to SNP markers generated from GBS, (UN) indicates an unknown genome.

## Slide 4
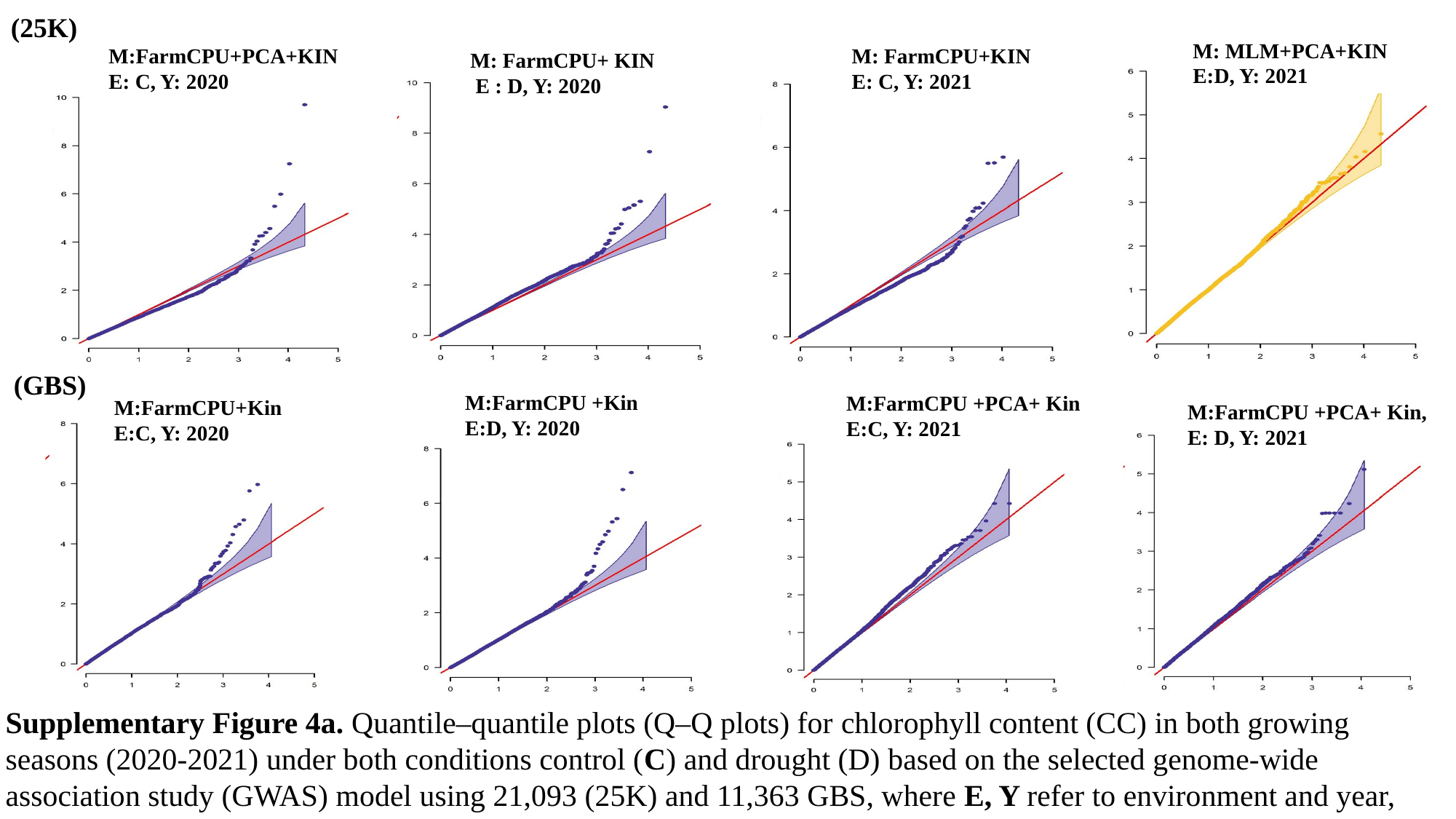

(25K)
M: MLM+PCA+KIN
E:D, Y: 2021
M: FarmCPU+KIN
E: C, Y: 2021
M:FarmCPU+PCA+KIN
E: C, Y: 2020
M: FarmCPU+ KIN
 E : D, Y: 2020
(GBS)
M:FarmCPU +Kin
E:D, Y: 2020
M:FarmCPU +PCA+ Kin
E:C, Y: 2021
M:FarmCPU+Kin
E:C, Y: 2020
M:FarmCPU +PCA+ Kin,
E: D, Y: 2021
Supplementary Figure 4a. Quantile–quantile plots (Q–Q plots) for chlorophyll content (CC) in both growing seasons (2020-2021) under both conditions control (C) and drought (D) based on the selected genome-wide association study (GWAS) model using 21,093 (25K) and 11,363 GBS, where E, Y refer to environment and year, respectively.

## Slide 5
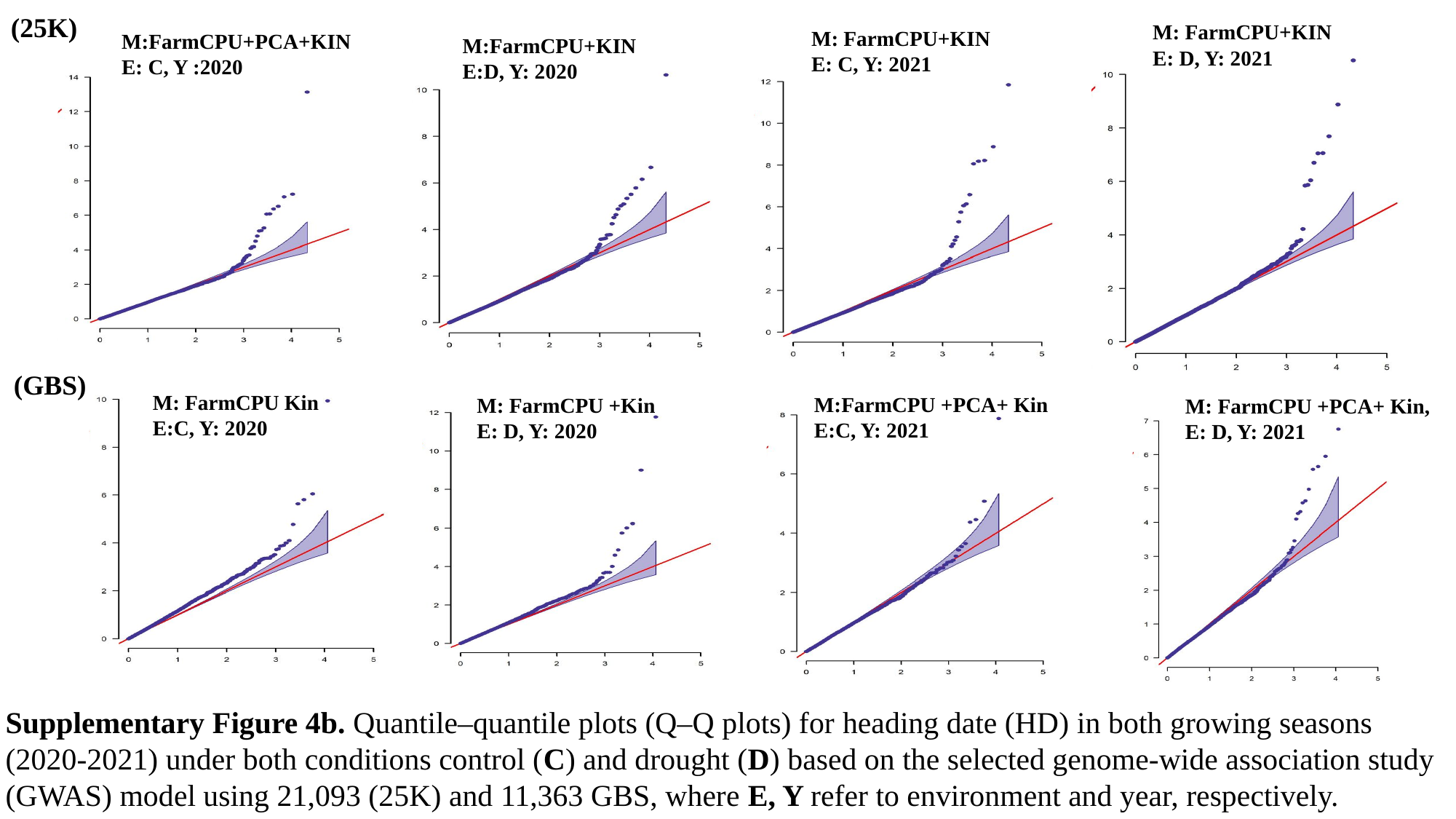

(25K)
M: FarmCPU+KIN
E: D, Y: 2021
M: FarmCPU+KIN
E: C, Y: 2021
M:FarmCPU+PCA+KIN
E: C, Y :2020
M:FarmCPU+KIN
E:D, Y: 2020
(GBS)
M: FarmCPU Kin
E:C, Y: 2020
M:FarmCPU +PCA+ Kin
E:C, Y: 2021
M: FarmCPU +Kin
E: D, Y: 2020
M: FarmCPU +PCA+ Kin,
E: D, Y: 2021
Supplementary Figure 4b. Quantile–quantile plots (Q–Q plots) for heading date (HD) in both growing seasons (2020-2021) under both conditions control (C) and drought (D) based on the selected genome-wide association study (GWAS) model using 21,093 (25K) and 11,363 GBS, where E, Y refer to environment and year, respectively.

## Slide 6
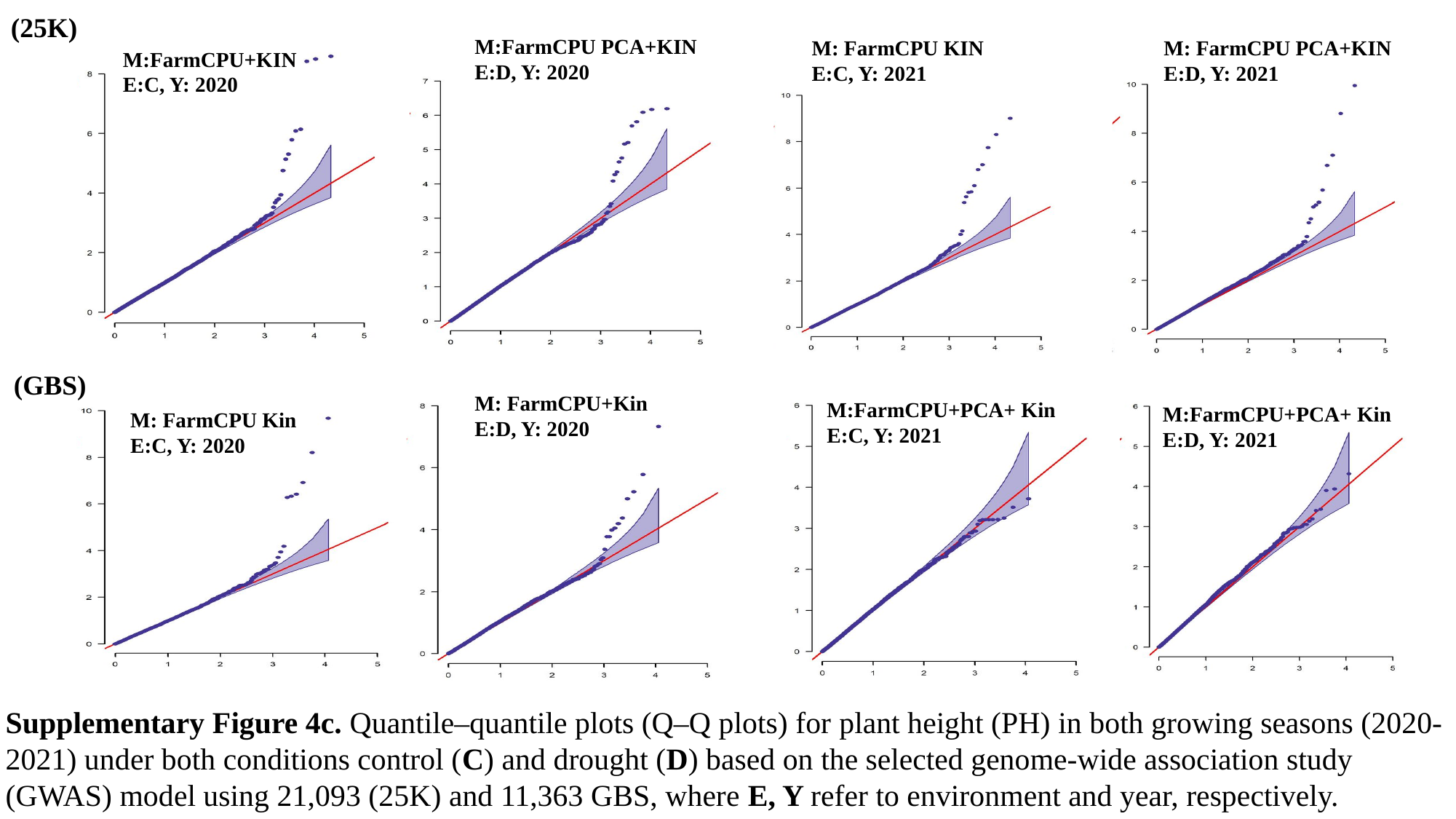

(25K)
M:FarmCPU PCA+KIN
E:D, Y: 2020
M: FarmCPU KIN
E:C, Y: 2021
M: FarmCPU PCA+KIN
E:D, Y: 2021
M:FarmCPU+KIN
E:C, Y: 2020
(GBS)
M: FarmCPU+Kin
E:D, Y: 2020
M:FarmCPU+PCA+ Kin
E:C, Y: 2021
M:FarmCPU+PCA+ Kin
E:D, Y: 2021
M: FarmCPU Kin
E:C, Y: 2020
Supplementary Figure 4c. Quantile–quantile plots (Q–Q plots) for plant height (PH) in both growing seasons (2020-2021) under both conditions control (C) and drought (D) based on the selected genome-wide association study (GWAS) model using 21,093 (25K) and 11,363 GBS, where E, Y refer to environment and year, respectively.

## Slide 7
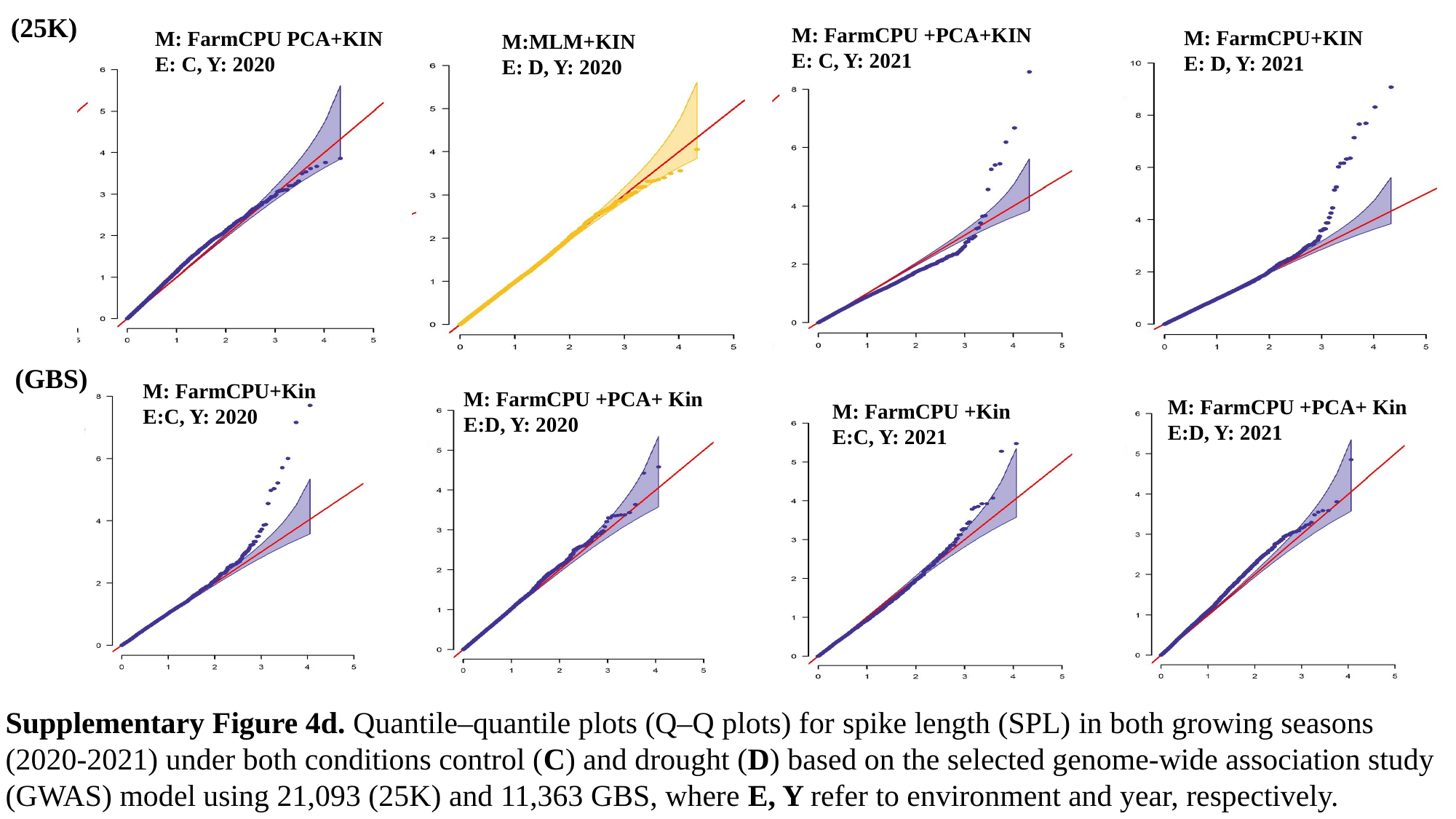

(25K)
M: FarmCPU +PCA+KIN
E: C, Y: 2021
M: FarmCPU+KIN
E: D, Y: 2021
M: FarmCPU PCA+KIN
E: C, Y: 2020
M:MLM+KIN
E: D, Y: 2020
(GBS)
M: FarmCPU+Kin E:C, Y: 2020
M: FarmCPU +PCA+ Kin
E:D, Y: 2020
M: FarmCPU +PCA+ Kin
E:D, Y: 2021
M: FarmCPU +Kin
E:C, Y: 2021
Supplementary Figure 4d. Quantile–quantile plots (Q–Q plots) for spike length (SPL) in both growing seasons (2020-2021) under both conditions control (C) and drought (D) based on the selected genome-wide association study (GWAS) model using 21,093 (25K) and 11,363 GBS, where E, Y refer to environment and year, respectively.

## Slide 8
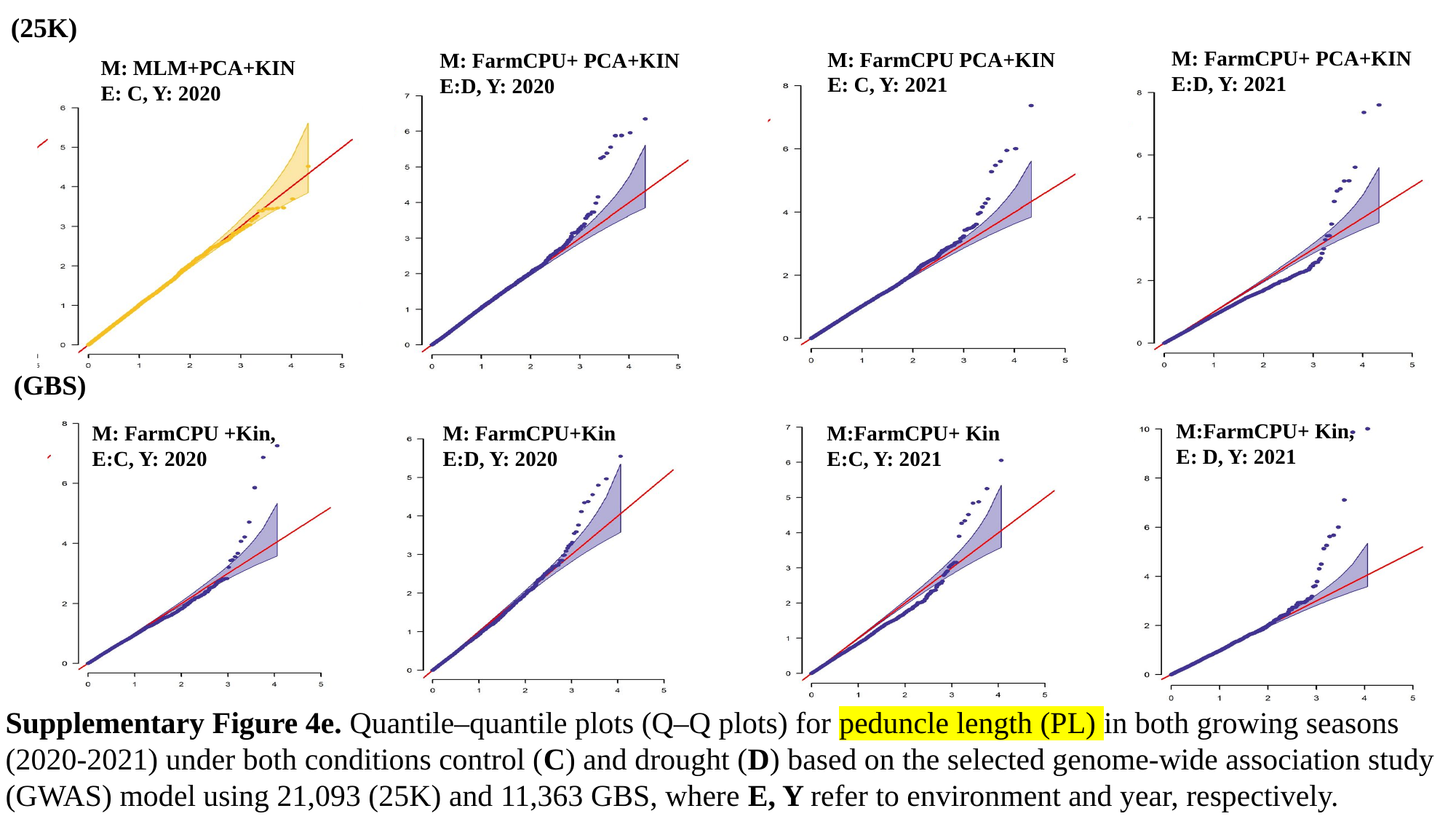

(25K)
M: FarmCPU+ PCA+KIN
E:D, Y: 2021
M: FarmCPU PCA+KIN
E: C, Y: 2021
M: FarmCPU+ PCA+KIN
E:D, Y: 2020
M: MLM+PCA+KIN
E: C, Y: 2020
(GBS)
M:FarmCPU+ Kin,
E: D, Y: 2021
M: FarmCPU +Kin,
E:C, Y: 2020
M: FarmCPU+Kin
E:D, Y: 2020
M:FarmCPU+ Kin
E:C, Y: 2021
Supplementary Figure 4e. Quantile–quantile plots (Q–Q plots) for peduncle length (PL) in both growing seasons (2020-2021) under both conditions control (C) and drought (D) based on the selected genome-wide association study (GWAS) model using 21,093 (25K) and 11,363 GBS, where E, Y refer to environment and year, respectively.

## Slide 9
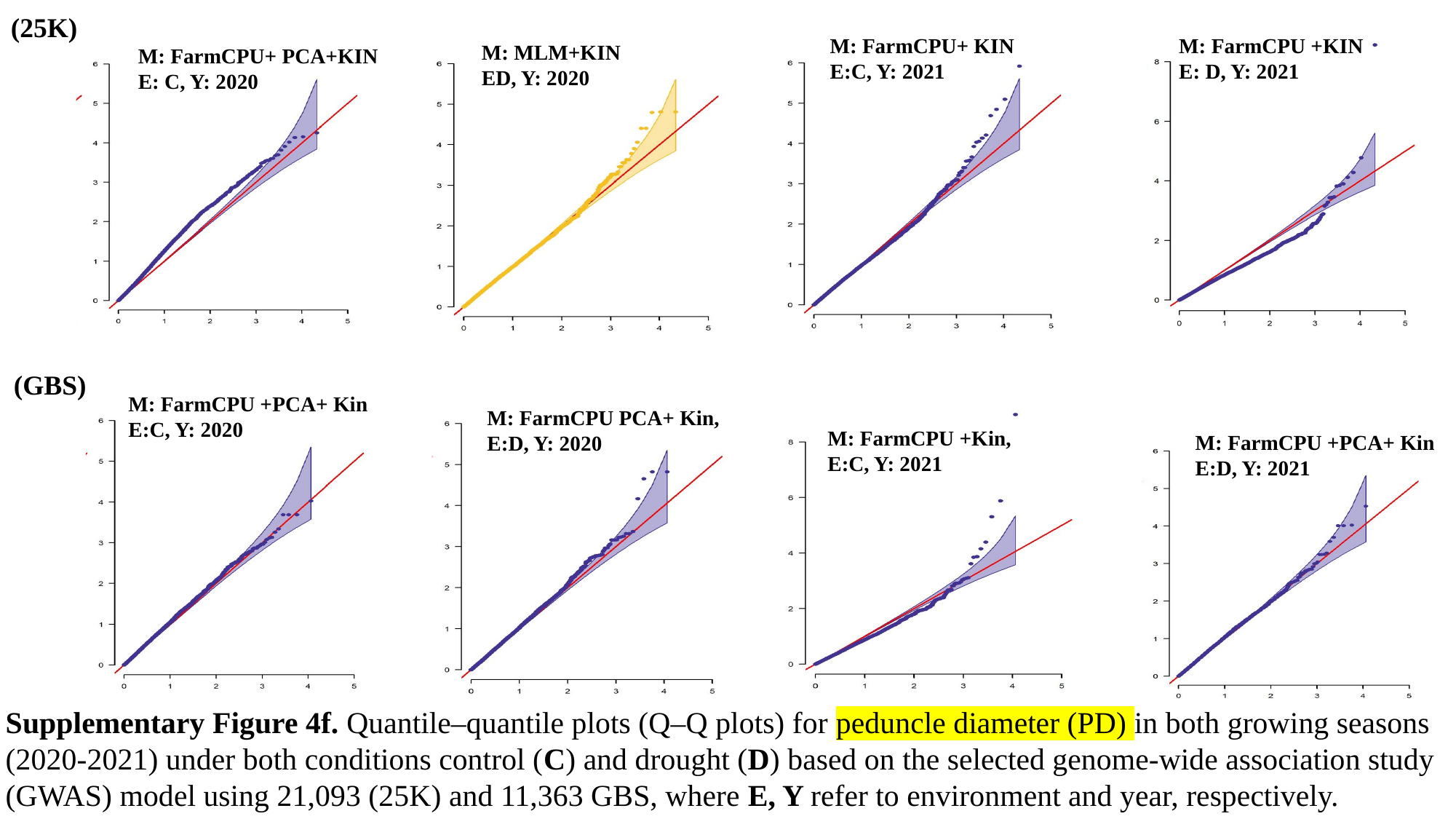

(25K)
M: FarmCPU+ KIN
E:C, Y: 2021
M: FarmCPU +KIN
E: D, Y: 2021
M: MLM+KIN
ED, Y: 2020
M: FarmCPU+ PCA+KIN
E: C, Y: 2020
(GBS)
M: FarmCPU +PCA+ Kin
E:C, Y: 2020
M: FarmCPU PCA+ Kin, E:D, Y: 2020
M: FarmCPU +Kin, E:C, Y: 2021
M: FarmCPU +PCA+ Kin
E:D, Y: 2021
Supplementary Figure 4f. Quantile–quantile plots (Q–Q plots) for peduncle diameter (PD) in both growing seasons (2020-2021) under both conditions control (C) and drought (D) based on the selected genome-wide association study (GWAS) model using 21,093 (25K) and 11,363 GBS, where E, Y refer to environment and year, respectively.

## Slide 10
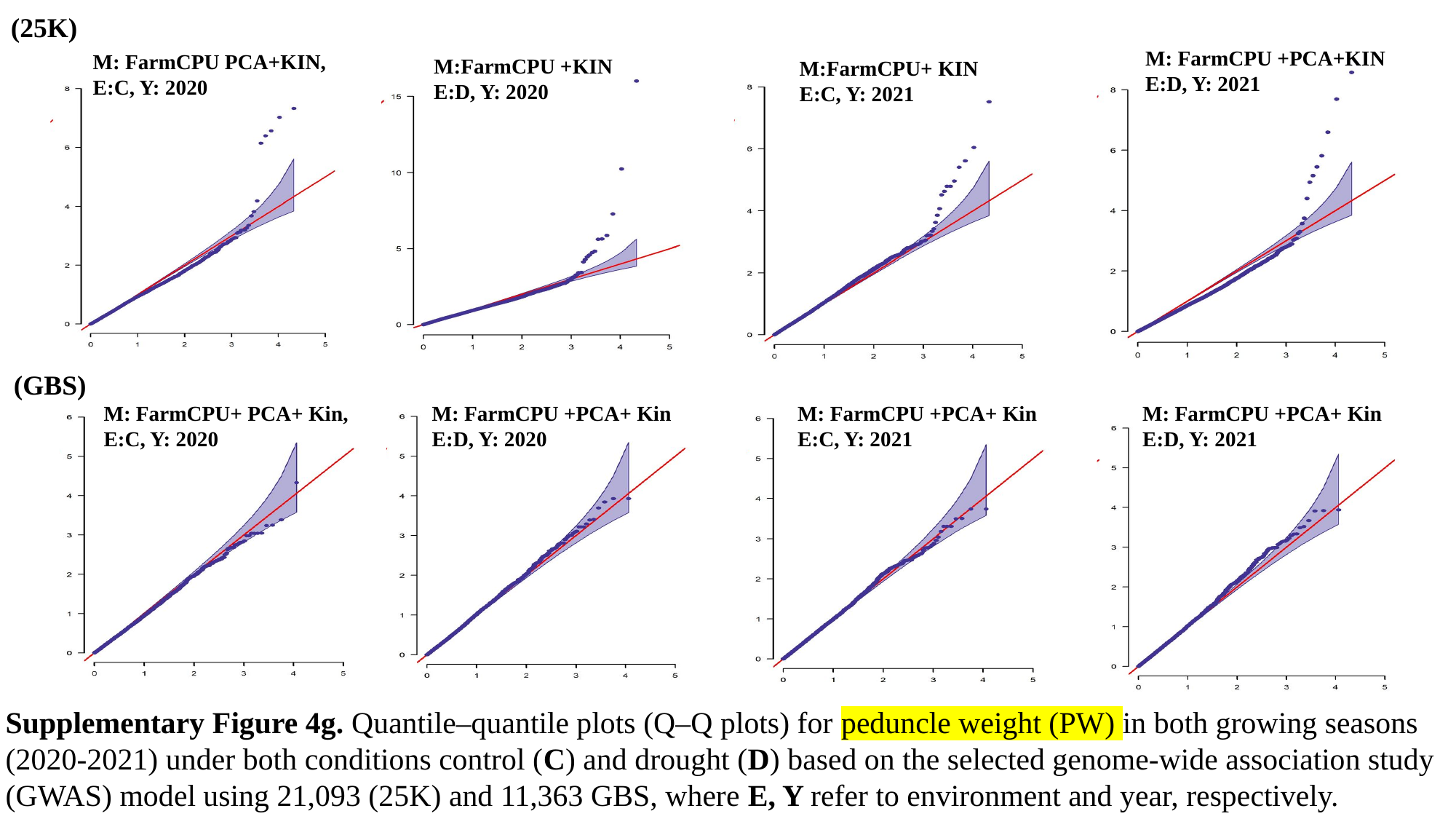

(25K)
M: FarmCPU +PCA+KIN
E:D, Y: 2021
M: FarmCPU PCA+KIN,
E:C, Y: 2020
M:FarmCPU +KIN
E:D, Y: 2020
M:FarmCPU+ KIN
E:C, Y: 2021
(GBS)
M: FarmCPU+ PCA+ Kin, E:C, Y: 2020
M: FarmCPU +PCA+ Kin
E:D, Y: 2020
M: FarmCPU +PCA+ Kin
E:C, Y: 2021
M: FarmCPU +PCA+ Kin
E:D, Y: 2021
Supplementary Figure 4g. Quantile–quantile plots (Q–Q plots) for peduncle weight (PW) in both growing seasons (2020-2021) under both conditions control (C) and drought (D) based on the selected genome-wide association study (GWAS) model using 21,093 (25K) and 11,363 GBS, where E, Y refer to environment and year, respectively.

## Slide 11
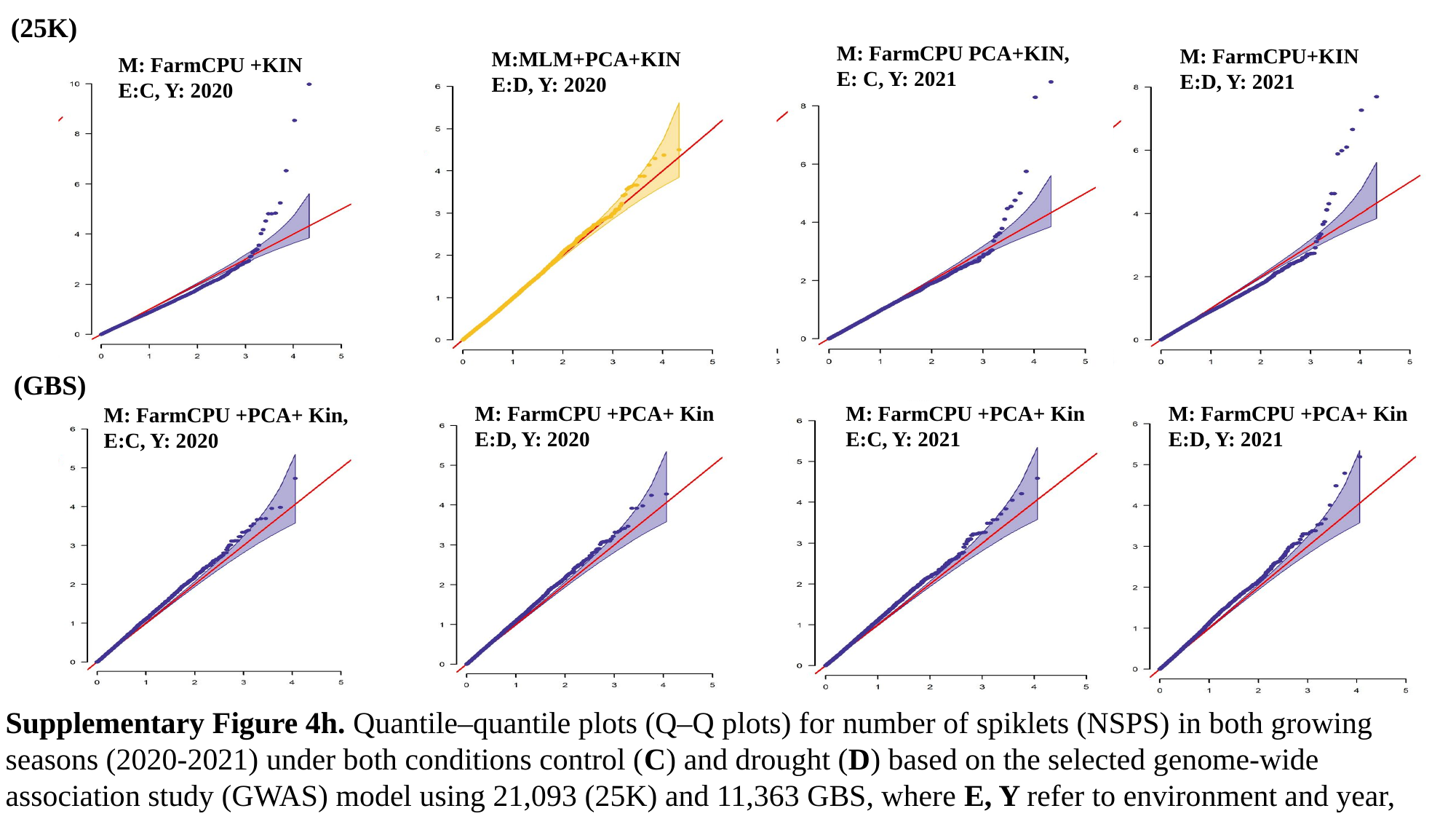

(25K)
M: FarmCPU PCA+KIN,
E: C, Y: 2021
M: FarmCPU+KIN
E:D, Y: 2021
M:MLM+PCA+KIN
E:D, Y: 2020
M: FarmCPU +KIN
E:C, Y: 2020
(GBS)
M: FarmCPU +PCA+ Kin
E:C, Y: 2021
M: FarmCPU +PCA+ Kin E:D, Y: 2021
M: FarmCPU +PCA+ Kin
E:D, Y: 2020
M: FarmCPU +PCA+ Kin,
E:C, Y: 2020
Supplementary Figure 4h. Quantile–quantile plots (Q–Q plots) for number of spiklets (NSPS) in both growing seasons (2020-2021) under both conditions control (C) and drought (D) based on the selected genome-wide association study (GWAS) model using 21,093 (25K) and 11,363 GBS, where E, Y refer to environment and year, respectively

## Slide 12
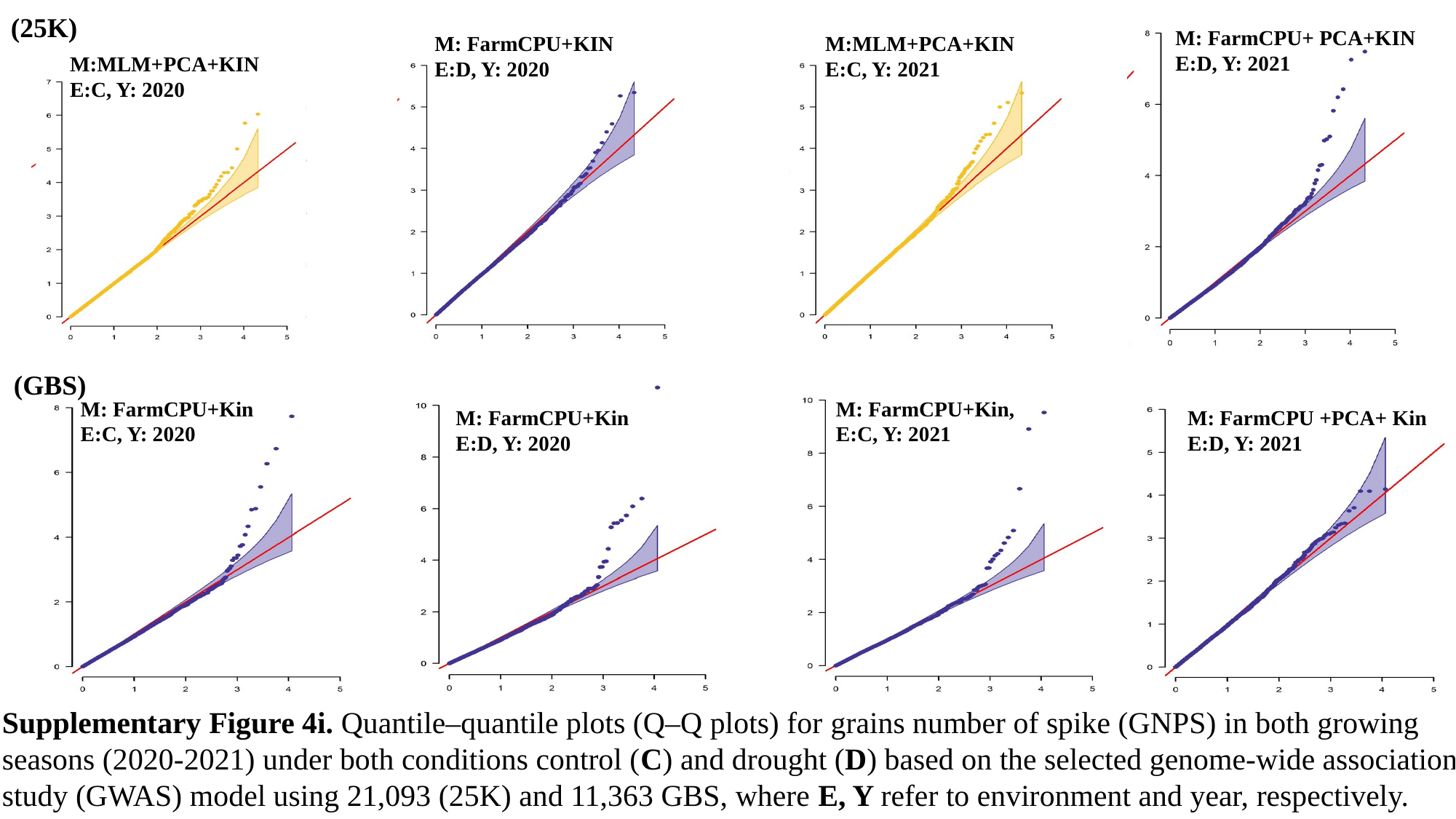

(25K)
M: FarmCPU+ PCA+KIN
E:D, Y: 2021
M: FarmCPU+KIN
E:D, Y: 2020
M:MLM+PCA+KIN
E:C, Y: 2021
M:MLM+PCA+KIN
E:C, Y: 2020
(GBS)
M: FarmCPU+Kin
E:C, Y: 2020
M: FarmCPU+Kin, E:C, Y: 2021
M: FarmCPU+Kin
E:D, Y: 2020
M: FarmCPU +PCA+ Kin
E:D, Y: 2021
Supplementary Figure 4i. Quantile–quantile plots (Q–Q plots) for grains number of spike (GNPS) in both growing seasons (2020-2021) under both conditions control (C) and drought (D) based on the selected genome-wide association study (GWAS) model using 21,093 (25K) and 11,363 GBS, where E, Y refer to environment and year, respectively.

## Slide 13
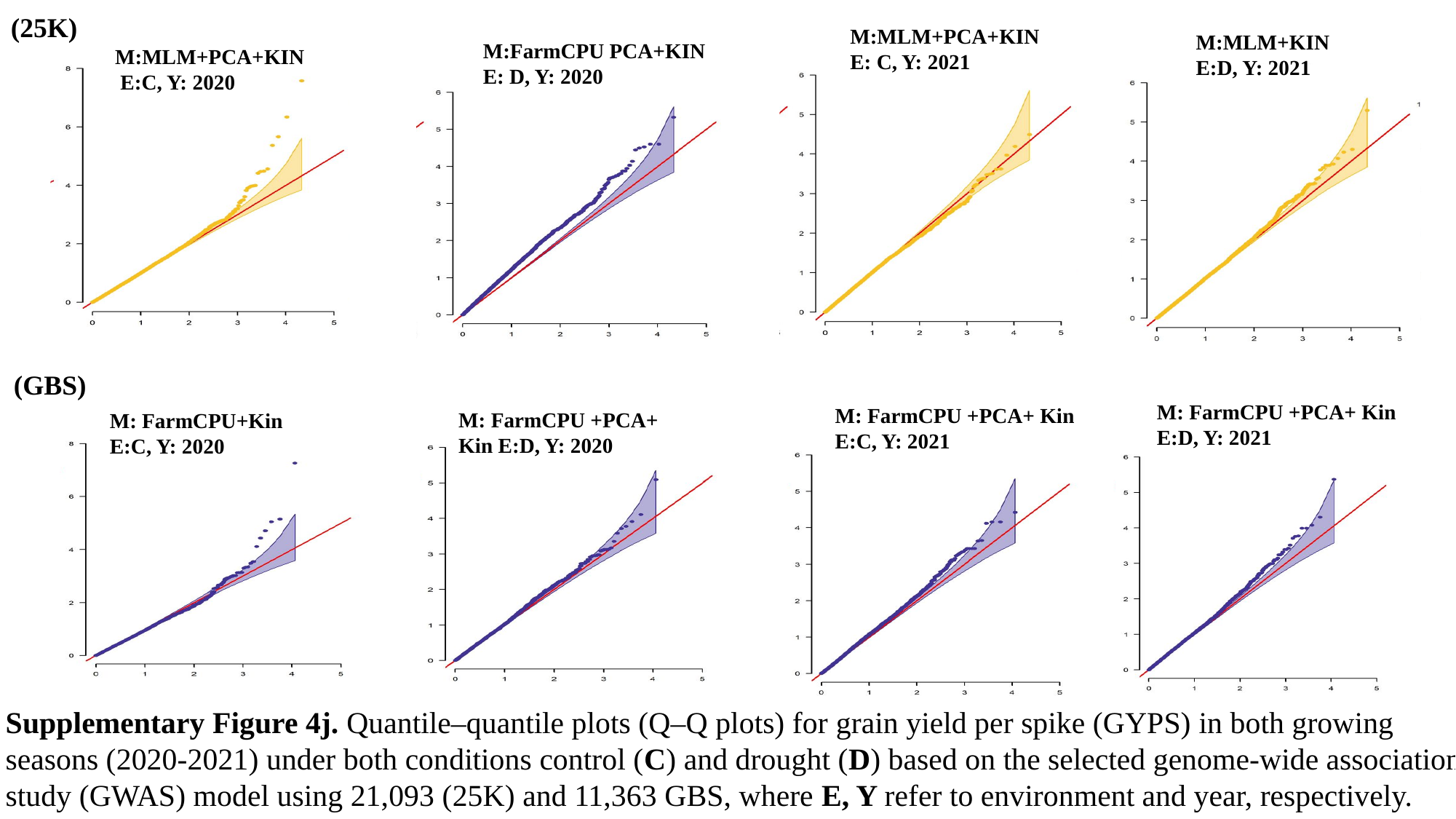

(25K)
M:MLM+PCA+KIN
E: C, Y: 2021
M:MLM+KIN
E:D, Y: 2021
M:FarmCPU PCA+KIN
E: D, Y: 2020
M:MLM+PCA+KIN
 E:C, Y: 2020
(GBS)
M: FarmCPU +PCA+ Kin
E:D, Y: 2021
M: FarmCPU +PCA+ Kin
E:C, Y: 2021
M: FarmCPU +PCA+ Kin E:D, Y: 2020
M: FarmCPU+Kin
E:C, Y: 2020
Supplementary Figure 4j. Quantile–quantile plots (Q–Q plots) for grain yield per spike (GYPS) in both growing seasons (2020-2021) under both conditions control (C) and drought (D) based on the selected genome-wide association study (GWAS) model using 21,093 (25K) and 11,363 GBS, where E, Y refer to environment and year, respectively.

## Slide 14
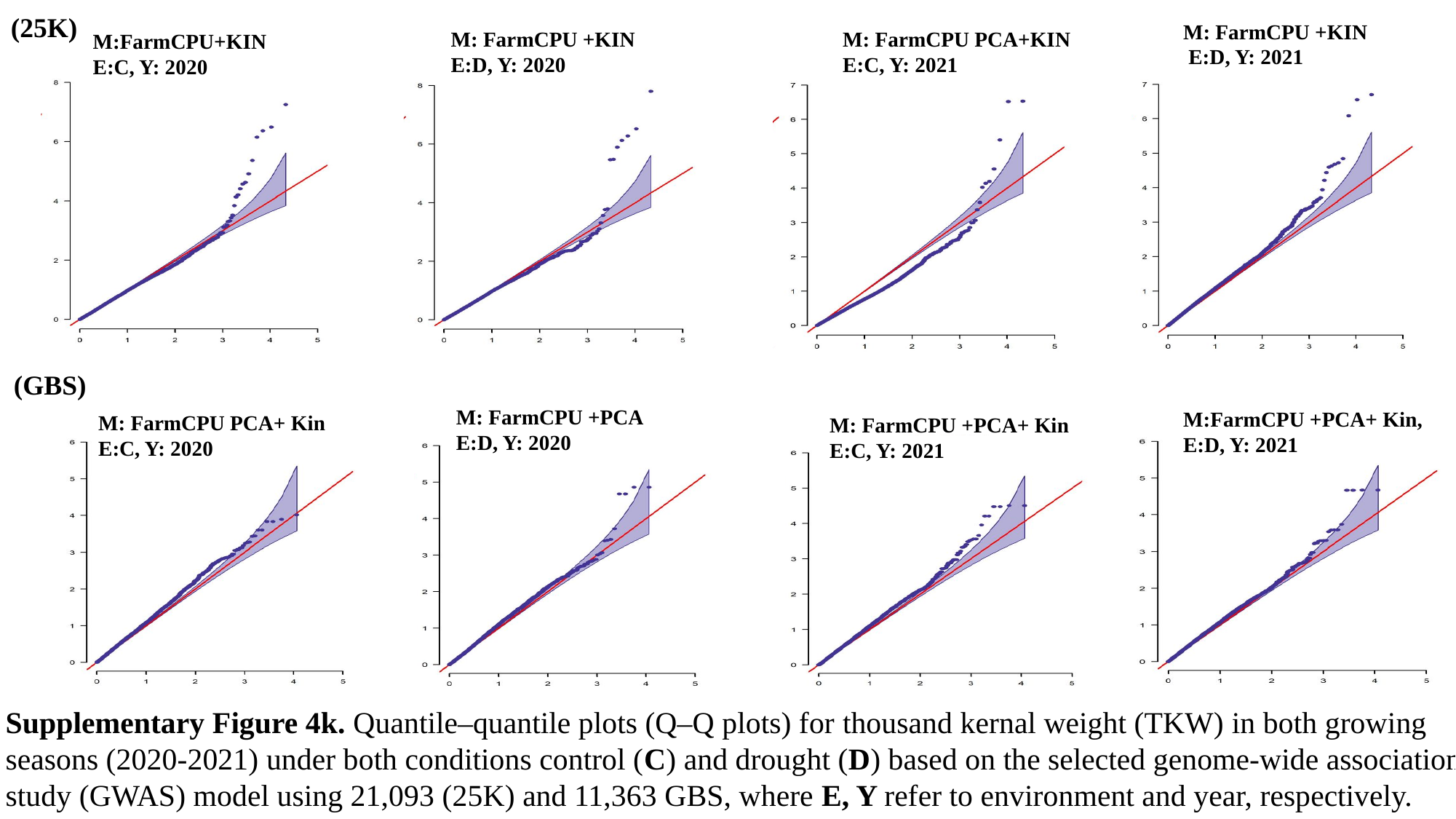

(25K)
M: FarmCPU +KIN
 E:D, Y: 2021
M: FarmCPU +KIN
E:D, Y: 2020
M: FarmCPU PCA+KIN
E:C, Y: 2021
M:FarmCPU+KIN
E:C, Y: 2020
(GBS)
M: FarmCPU +PCA
E:D, Y: 2020
M:FarmCPU +PCA+ Kin,
E:D, Y: 2021
M: FarmCPU PCA+ Kin
E:C, Y: 2020
M: FarmCPU +PCA+ Kin
E:C, Y: 2021
Supplementary Figure 4k. Quantile–quantile plots (Q–Q plots) for thousand kernal weight (TKW) in both growing seasons (2020-2021) under both conditions control (C) and drought (D) based on the selected genome-wide association study (GWAS) model using 21,093 (25K) and 11,363 GBS, where E, Y refer to environment and year, respectively.

## Slide 15
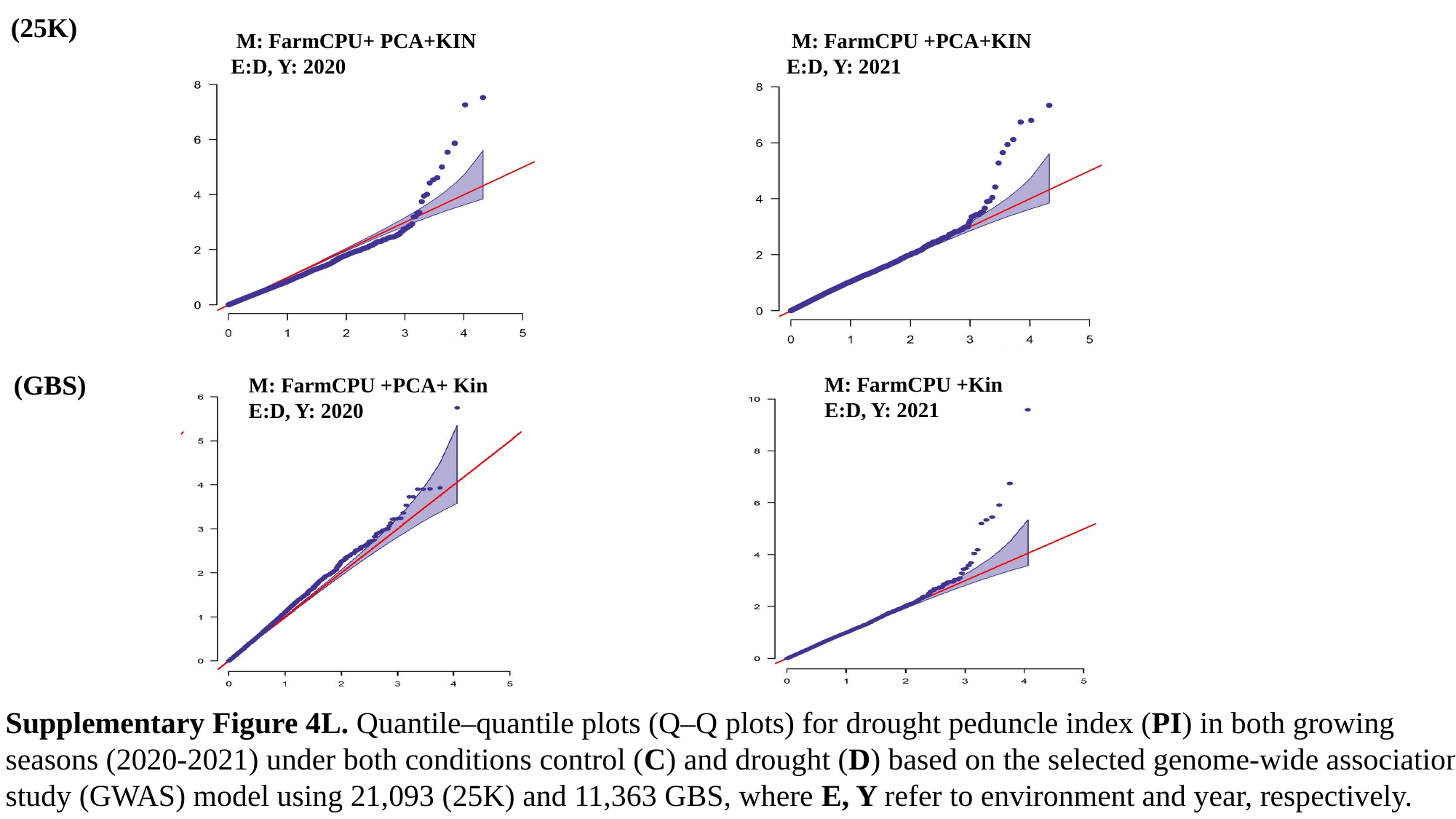

(25K)
 M: FarmCPU +PCA+KIN
E:D, Y: 2021
 M: FarmCPU+ PCA+KIN
E:D, Y: 2020
(GBS)
M: FarmCPU +Kin
E:D, Y: 2021
M: FarmCPU +PCA+ Kin
E:D, Y: 2020
Supplementary Figure 4L. Quantile–quantile plots (Q–Q plots) for drought peduncle index (PI) in both growing seasons (2020-2021) under both conditions control (C) and drought (D) based on the selected genome-wide association study (GWAS) model using 21,093 (25K) and 11,363 GBS, where E, Y refer to environment and year, respectively.

## Slide 16
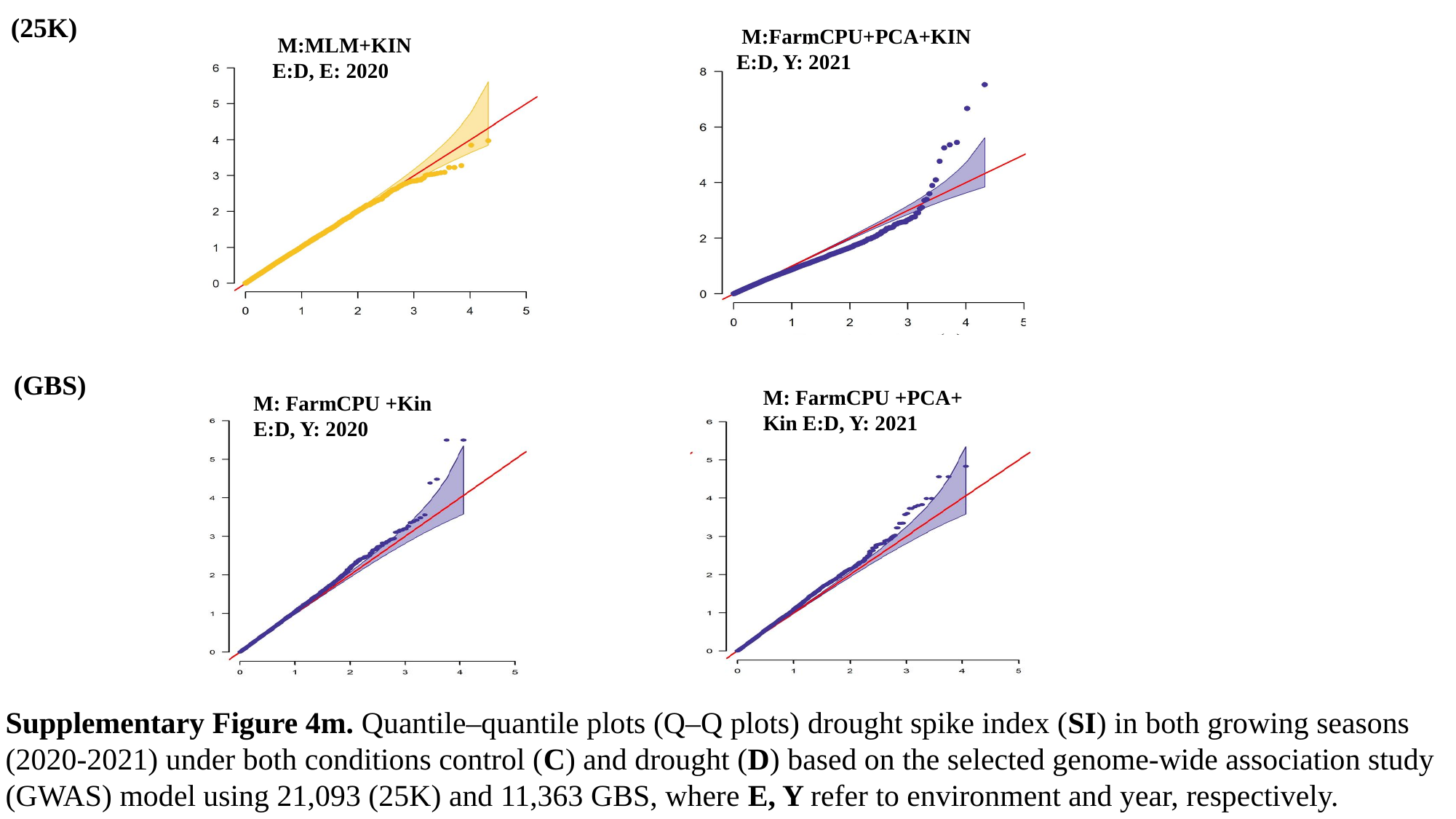

(25K)
 M:FarmCPU+PCA+KIN
E:D, Y: 2021
 M:MLM+KIN
E:D, E: 2020
(GBS)
M: FarmCPU +PCA+ Kin E:D, Y: 2021
M: FarmCPU +Kin
E:D, Y: 2020
Supplementary Figure 4m. Quantile–quantile plots (Q–Q plots) drought spike index (SI) in both growing seasons (2020-2021) under both conditions control (C) and drought (D) based on the selected genome-wide association study (GWAS) model using 21,093 (25K) and 11,363 GBS, where E, Y refer to environment and year, respectively.

## Slide 17
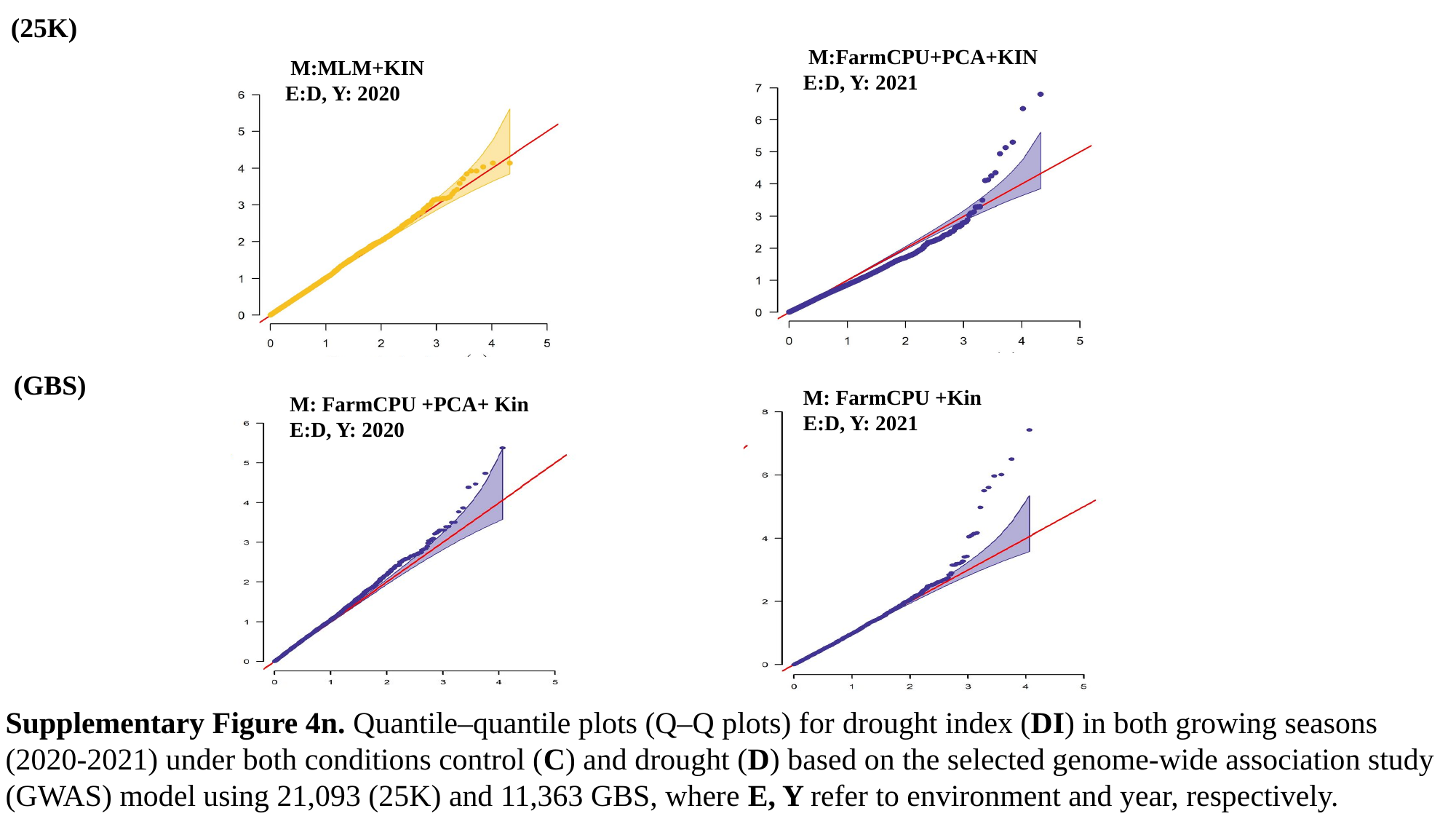

(25K)
 M:FarmCPU+PCA+KIN
E:D, Y: 2021
 M:MLM+KIN
E:D, Y: 2020
(GBS)
M: FarmCPU +Kin
E:D, Y: 2021
M: FarmCPU +PCA+ Kin
E:D, Y: 2020
Supplementary Figure 4n. Quantile–quantile plots (Q–Q plots) for drought index (DI) in both growing seasons (2020-2021) under both conditions control (C) and drought (D) based on the selected genome-wide association study (GWAS) model using 21,093 (25K) and 11,363 GBS, where E, Y refer to environment and year, respectively.
